# Mechanisms by which microbial enzymes degrade four mycotoxins and application in animal production: A review

**DOI:** 10.1016/j.aninu.2023.09.003

**Published:** 2023-10-04

**Authors:** Huiying Sun, Ziqi He, Dongwei Xiong, Miao Long

**Affiliations:** a Key Laboratory of Livestock Infectious Diseases, Ministry of Education, Key Laboratory of Ruminant Infectious Disease Prevention and Control (East), Ministry of Agriculture and Rural Affairs, China; b College of Animal Science and Veterinary Medicine, Shenyang Agricultural University, Shenyang, 110866, China

**Keywords:** Mechanism, Microbial enzyme, Degradation, Mycotoxin

## Abstract

Mycotoxins are toxic compounds that pose a serious threat to animal health and food safety. Therefore, there is an urgent need for safe and efficient methods of detoxifying mycotoxins. As biotechnology has continued to develop, methods involving biological enzymes have shown great promise. Biological enzymatic methods, which can fundamentally destroy the structures of mycotoxins and produce degradation products whose toxicity is greatly reduced, are generally more specific, efficient, and environmentally friendly. Mycotoxin-degrading enzymes can thus facilitate the safe and effective detoxification of mycotoxins which gives them a huge advantage over other methods. This article summarizes the newly discovered degrading enzymes that can degrade four common mycotoxins (aflatoxins, zearalenone, deoxynivalenol, and ochratoxin A) in the past five years, and reveals the degradation mechanism of degrading enzymes on four mycotoxins, as well as their positive effects on animal production. This review will provide a theoretical basis for the safe treatment of mycotoxins by using biological enzyme technology.

## 1 Introduction

Mycotoxins are secondary metabolites produced by fungi such as *Aspergillus*, *Penicillium*, and *Fusarium*. They are widely found in mildew on plants, in food, and in feed, and are generally difficult to remove. Direct and indirect contact can cause poisoning in humans and animals (Skrzydlewski et al., 2022). Based on data and an analysis of the available literature, the current prevalence of mycotoxins in food crops worldwide appears to be as high as 60% to 80%; climate change may also have played a role in their higher incidence and will only increase this level of pollution in the future (Eskola et al., 2020).

Up to now, more than 400 mycotoxins have been identified in the world. Among these, the most commonly encountered mycotoxins are ochratoxin A (OTA), aflatoxins, zearalenone (ZEN), deoxynivalenol (DON), and fumonisins (Karlovsky et al., 2016). The harmful effects of mycotoxins are numerous and include reproductive toxicity, immunotoxicity, genetic toxicity, hepatorenal toxicity, and cardiac toxicity (Dai et al., 2022a; L. Li et al., 2021; Pleadin et al., 2019; Sun et al., 2022; Ulger et al., 2020). In addition, mycotoxin exposure is closely related to the occurrence of cancer (Cao et al., 2022; Singh et al., 2022). To date, mycotoxins with carcinogenic potency as reported in the literature include aflatoxins, ochratoxins, fumonisins, ZEN, and some *Penicillium* toxins. Although DON is not considered carcinogenic to humans, its regulation of ROS production in tumor cells may indirectly assist the progression of tumors via apoptosis (Cao et al., 2022; Singh et al., 2022).

Mycotoxicosis caused by improper feed storage (wet or high-temperature conditions) is often less easily diagnosed compared to other diseases, thus missing the optimal time for treatment. Moreover, most mycotoxins coexist in the external environment and there are likely to be new mycotoxins that have not yet been detected. This can further aggravate their harmful effects and makes them an even greater threat to the health of human beings and animals (Miguel et al., 2022; Mutiga et al., 2021; Zhang et al., 2022). Therefore, reducing mycotoxin pollution is one of the main tasks of the agriculture and food industries.

A variety of physical and chemical detoxification methods have been found to solve the problem of mycotoxin contamination. Each of which has been successful to a certain extent. Physical methods include adsorption, peeling, grinding and cleaning, high-temperature heating, irradiation, low-temperature ion cooling, and ozone oxidation (Jard et al., 2011; Yousefi et al., 2021). However, it is difficult to completely remove toxins using physical methods. Furthermore, most of the traditional adsorbents that have a good adsorption effect with respect to aflatoxins demonstrate a low adsorption capacity towards ZEN, DON, T-2 toxin, and other mycotoxins which can limit their use (Kihal et al., 2022).

Chemical detoxification methods involve introducing chemical reagents to cause ammoniation, oxidation, reduction, hydrolysis, and other chemical reactions in order to reduce the toxicity of the mycotoxin. However, the residual chemicals used in such methods may destroy the nutrients in the crops which will not only seriously affect the nutritional value and taste of the food but can also exert secondary toxic effects and pollute the environment (S. Li et al., 2018). It can thus be found that physical and chemical methods cannot completely solve the problem of mycotoxin contamination.

In recent years, biological detoxification methods (including microbial adsorption and microbial degradation) have started to become more popular compared to physical and chemical methods (Agriopoulou et al., 2020). Such methods are safer and more efficient at detoxifying mycotoxins and also protect the nutritional value of the food and feed. Microbial adsorption refers to the use of microbes to adsorb or metabolize toxins to realize detoxification. The removal effect achieved is very significant but such methods are not applicable to all fields (e.g. the food processing process) (N. Wang et al., 2019).

Microbial degradation refers to the use of active substances secreted by microorganisms to change the original structures of mycotoxins. The mycotoxins are thereby transformed into products that are less toxic or even not toxic at all (Guan et al., 2021). Compared to the use of live microorganisms, enzyme degradation has certain advantages, namely, treatment is easy, highly repeatable, uniform in performance, highly efficient, and high in specificity. Furthermore, treatment can be conducted using mild conditions (optimal temperature and pH value) and there is a much smaller reduction in levels of dietary nutrients (Ndiaye et al., 2022). In addition, some studies have found that biological enzymes can also reduce the body damage caused by mycotoxins (Prasad et al., 2023; Danicke et al., 2023; Li et al., 2019). Therefore, mycotoxin-degrading enzymes have good development prospects for the future. However, it is necessary to understand the structure, degradation pathway, and products of mycotoxin-degrading enzymes when they are used.

The common degradation enzymes of four mycotoxins (aflatoxins, ZEN, OTA, and DON) and their degradation pathways are summarized, as well as the possible metabolites of each step, providing a reliable reference strategy for the degradation of four mycotoxins in food and feed by bioenzymes and their practical application.

## 2 Aflatoxins

Aflatoxins are secondary metabolites produced by *Aspergillus flavus* and *A. parasiticus*. They include aflatoxin B1 (AFB1), aflatoxin B2 (AFB2), aflatoxin G1 (AFG1), aflatoxin G2 (AFG2), aflatoxin M1 (AFM1), and aflatoxin M2 (AFM2) (Jiang et al., 2021). Among these, the most toxic is AFB1. Aflatoxins have high carcinogenicity, teratogenicity, hepatorenal toxicity, and immunotoxicity (Cao et al., 2022; D. Gao et al., 2022; Hua et al., 2020; Yilmaz et al., 2018). They can result in cytotoxicity in various ways, such as: intracellular accumulation of reactive oxygen species, oxidative stress, lipid peroxidation, mitochondrial dysfunction, autophagy, and apoptosis (Dai et al., 2022b). In addition, aflatoxins can further affect the energy supply of the body by disrupting the metabolic pathways of the gut microbiota, thus leading to certain metabolic diseases (Zhou et al., 2018). Mothers exposed to aflatoxins can also have the intestinal barrier homeostasis of their offspring directly affected which reduces their ability to resist intestinal pathogens (Bastos-Amador et al., 2023).

**Fig. 1:**
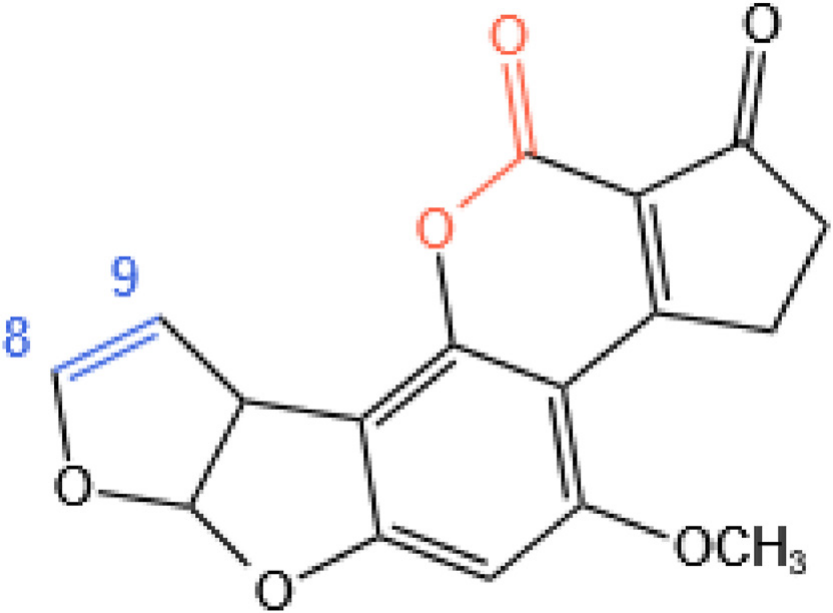
The structure responsible for the toxicity of aflatoxins. The lactone bond is marked in orange and the double bond is marked in blue.

**Fig. 2:**
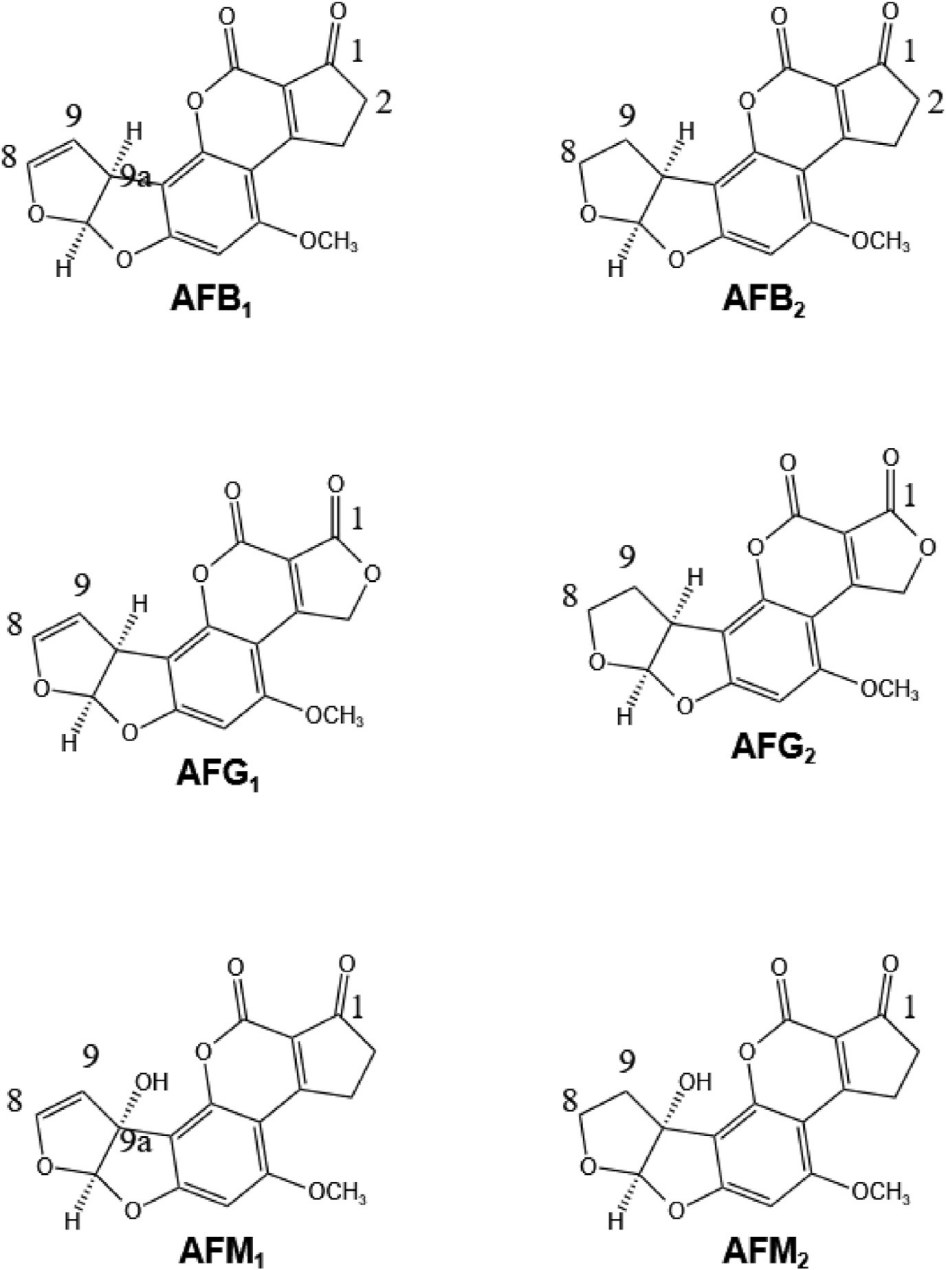
The structures of some natural aflatoxins. Aflatoxin B1, B2, G1, G2, M1, and M2 have pairwise structural differences at opposite moieties of the molecules with aflatoxin 1 and 2 differing in the 8,9-positions on one side, aflatoxin B, M and G differing in the 1-position on the other side as well as aflatoxin B, G, and M differing in the 9a-position.

The toxicity of aflatoxins is related to the C8–C9 double bond in the difuran ring system and lactone ring in the coumarin ring system present in their molecules, as shown in Fig. 1. The molecular structures of six naturally-occurring aflatoxins are illustrated in Fig. 2.

### 2.1 Enzymatic degradation of AFB1

A variety of enzymes have been reported to degrade aflatoxins. Table 1 presents the aflatoxin B1-degrading enzymes from different microbial sources which have been found in the past five years. These findings provide some new references suggesting that microbial enzymes can be used to degrade the mycotoxins present in food and feed.

**Table 1:** Aflatoxin-degrading enzymes from different microbial sources.

Enzyme abbreviations	Sources	Enzyme names	Identified substrates	Degradation conditions and effects	References
LAC2	*Cerrena unicolor* 6884	Laccase	AFB1	The degradation efficiency was 94% at pH 7.0 and 45 °C for 24 h.	Zhou et al. (2020)
LAC3	*Trametes* sp. C30	Laccase	AFB1	The degradation efficiency was 90.33% at pH 5.7 and 30 °C for 24 h.	Liu et al. (2020)
			AFB2	The degradation efficiency was 74.23% at pH 5.7 and 30 °C for 72 h.	
			AFG1	The degradation efficiency was 85.24% at pH 5.7 and 30 °C for 48 h.	
			AFG2	The degradation efficiency was 87.58% at pH 5.7 and 30 °C for 48 h.	
Rh_DypB	*Escherichia coli* BL21(DE3)	Peroxidase	AFB1	At 25 °C and pH 6.0 for 4 d, at 2 mmol/L MnCl_2_, 0.1 mmol/L H_2_O_2_, the degradation efficiency was 96%.	Loi et al. (2020)
fmb-rL103	*Bacillus vallismortis* fmb-103	Laccase	AFB1	The degradation efficiency exceeded 60% at pH 7.0 and 37 °C.	Bian et al. (2022)
MSMEG_5998	*Mycobacterium smegmatis*	F_420_H_2_-dependent reductase	AFB1	The degradation efficiency was 80% at pH 7.4 and 22 °C for 8 h.	Li et al. (2019)
NR^1^	*Pseudomonas putida*	Lipase	AFB1	The degradation efficiency exceeded 80% at pH 7.0 and 37 °C for 4 h.	Singh and Mehta (2022)
BADE	*Bacillus shackletonii* L7	Oxidoreductase	AFB1	The 10 mmol/L Cu^2+^ can increase activity. At 70 °C and pH 8.0, the degradation efficiency was 47.15% for 72 h.	Xu et al. (2017)
PADE	*Pantoea* sp. T6	NR^1^	AFB1	The optimal activity conditions are 40 °C and pH 7.0.	Xie et al. (2019)
TV–AFB1D	*Trametes versicolor*	NR^1^	AFB1	The degradation efficiency was 67.40% at pH 7.0 and 32 °C for 5 d.	Yang et al. (2022)
*Bs*DyP	*Bacillus subtilis* SCK6	Peroxidase	AFB1	The degradation efficiency was 76.93% at pH 4.0 and 30 °C for 48 h.	Qin et al. (2021a)
PhcMnp	Recombinant *Kluyveromyces lactis* GG799 (pKLAC1-Phcmnp)	Manganese peroxidase	AFB1	The optimal degradation system contains 1.0 mmol/L MnSO4, 3.5 mmol/L glucose, and 1.2 U/mL glucose oxidase, pH 4.5 and 40 °C for 36 h.	Xia et al. (2021)
*St*MCO	*Streptomyces thermocarboxydus*	Multicopper oxidases	AFB1	At pH 7.0 and 30 °C for 24 h, at 1 mmol/L AS, the degradation efficiency was 99.85%.	Qin et al. (2021c)
				At pH 7.0 and 30 °C for 24 h, at 1 mmol/L SA, the degradation efficiency was 93.03%.	
				At pH 7.0 and 30 °C for 24 h, at 1 mmol/L ferulic acid, the degradation efficiency was 81.19%.	
*Bs*CotA	*Bacillus subtilis*	Laccase	AFB1	At pH 7.0 and 30 °C for 10h, at 1 mmol/L methyl syringate, the degradation efficiency was 98%.	Wang et al. (2019)
NR^1^	*Pseudomonas aeruginosa*	Catalase	AFB1	At SA, pH 7.4 and 37 °C, the degradation rate was 38.79% for 72 h.	Yanhua et al. (2023)

AF = aflatoxin; AS = acetosyringone; SA = syringaldehyde; MnSO_4_ = manganese sulfate; MnCl_2_ = manganese chloride; H_2_O_2_ = hydrogen peroxide.

1 NR denotes information that has not been reported.

### 2.2 Mechanisms of action of AFB1-degrading enzymes

AFB1 can be degraded by subjecting it to hydroxylation, epoxidation, reduction, or demethylation. As already mentioned, the two key sites affecting the toxicity of AFB1 are the furan and lactone ring structures. Aflatoxin-degrading enzymes therefore mainly act by changing these ring structures. However, further experiments are required to show that the degradation products generated are less toxic than the AFB1 itself (Guo et al., 2020; Loi et al., 2020).

#### 2.2.1 Changing the toxic lactone ring structure in AFB1

The lactone ring structure in AFB1 is easily hydrolyzed which makes it an easy target for toxin degradation. Gonzalez et al. (2019) amplified a 753 pb fragment from *Bacillus subtilis* RC1B, *B*. *cereus* RC1C, and *B*. *mojavensis* RC3B which degrades AFB1. The fragment was subsequently verified to correspond to the *aiiA* gene encoding *N*-acyl-homoserine lactonase. This finding implies that the lactone ring in AFB1 is the likely target of the degrading enzyme.

Guan et al. (2010) found that the ANSM068 strain of *Myxococcus flavus* bioconverted AFB1 into another compound with a different structure via an enzymatic reaction. High performance liquid chromatography and Fourier-transform infrared (IR) spectroscopy were used to confirm that the lactone ring attached to the benzene ring in AFB1 had been modified (for example, the characteristic IR absorption peak at 1,728 cm^−1^ in a standard AFB1 spectrum disappeared after treatment). Motomura et al. (2003) treated AFB1 with purified enzymes from *Pleurotus ostreatus* and found that the intensity of the fluorescence produced by the aflatoxin decreased significantly. Only a change in the lactone ring can cause such a change in the fluorescence intensity which suggests that the enzyme responsible had cracked open the lactone ring in the AFB1.

J. Li et al. (2018) utilized salt-tolerant *Candida universalis* CGMCC 3790 to degrade AFB1 and identified four nontoxic degradation products (C_14_H_10_O_4_, C_14_H_12_O_3_, C_13_H_12_O_2_, and C_11_H_10_O_4_). According to the structures of the products obtained, the authors inferred that the AFB1 had been degraded via two pathways. One pathway involves the lactone and benzene rings being hydrolyzed. A hydroxyl group is introduced between carbons 10 and 11 in AFB1 to directly open the lactone ring to produce a carboxylic acid group. Decarboxylation then occurs to produce the final products. The other pathway involves the ester bond and ether bond of the lactone ring being destroyed by hydrogenation. Kumar et al. (2023) also verified that the main site to target to reduce the toxicity of AFG1 is the lactone ring.

**Fig. 3:**
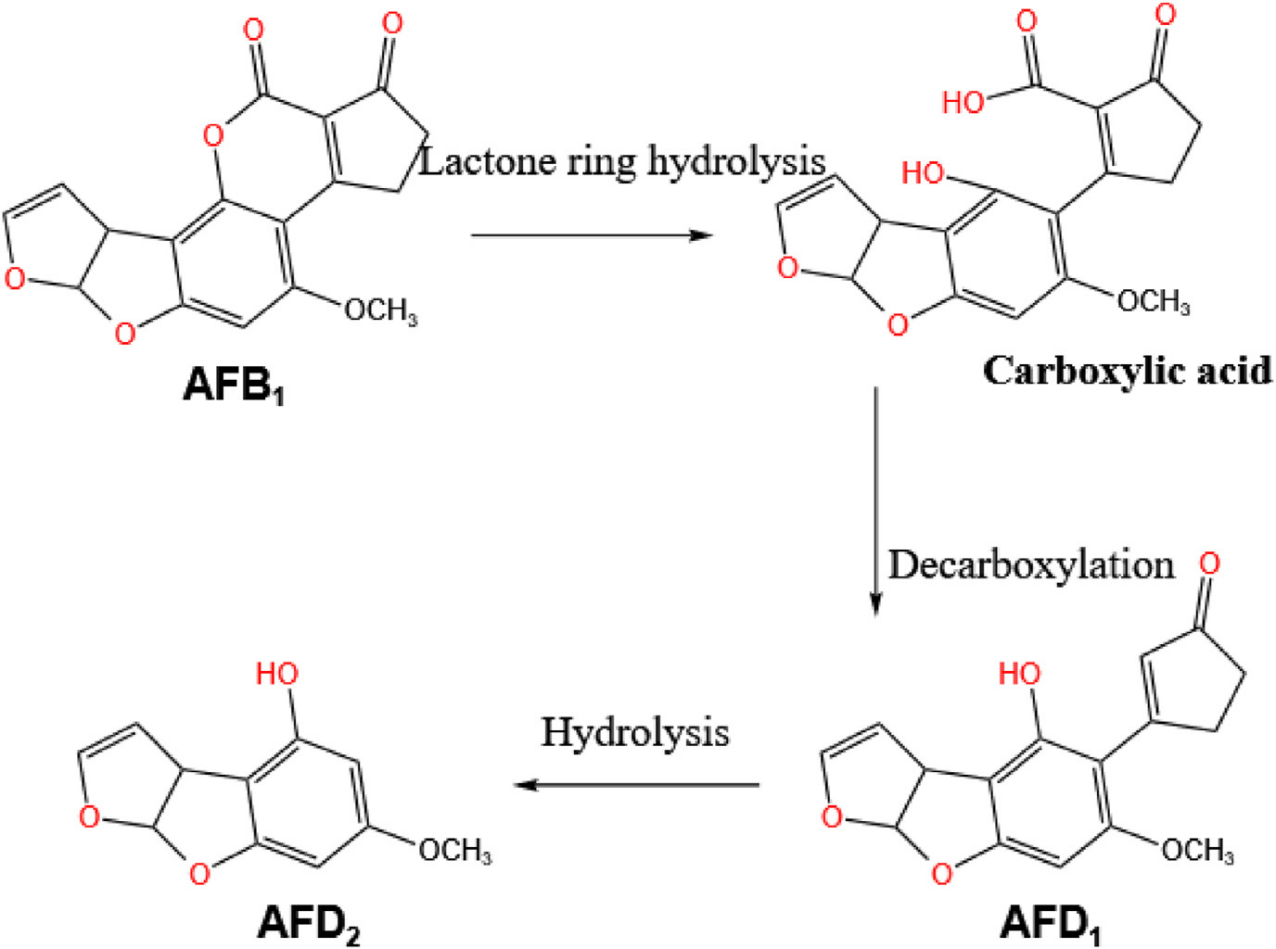
The mechanism of degradation of the AFB1 lactone ring. The lactone ring in AFB1 is opened by enzymatic hydrolysis to produce a carboxylic acid group which can then undergo decarboxylation and hydrolysis to produce AFD2. AFB1 = aflatoxin B1; AFD1 = aflatoxin D1; AFD2 = aflatoxin D2.

Some scholars have proposed that the redox enzyme can catalyze the hydrolysis of the lactone ring in AFB1 to produce a carboxylic acid group which can then undergo decarboxylation to produce aflatoxin D1 (AFD1), as illustrated in Fig. 3. Further hydrolysis can then lead to the cleavage of the bond to the cyclic peptide ring resulting in the formation of the derivative aflatoxin D2 (AFD2). Eshelli et al. (2015) further found that the intermediates produced by the hydrolysis of lactone rings have β-ketoic acid structures. It is worth mentioning that although the 8, 9-dihydrofuran double bond is retained in the final product AFD2, the lactonyl carbonyl group and cyclopentenone ring characteristics of the AFB1 molecule disappear and so the toxicities of the products are still much lower than that of AFB1.

#### 2.2.2 Changing the toxic 8, 9-dihydrofuran double bond structure in AFB1

**Fig. 4:**
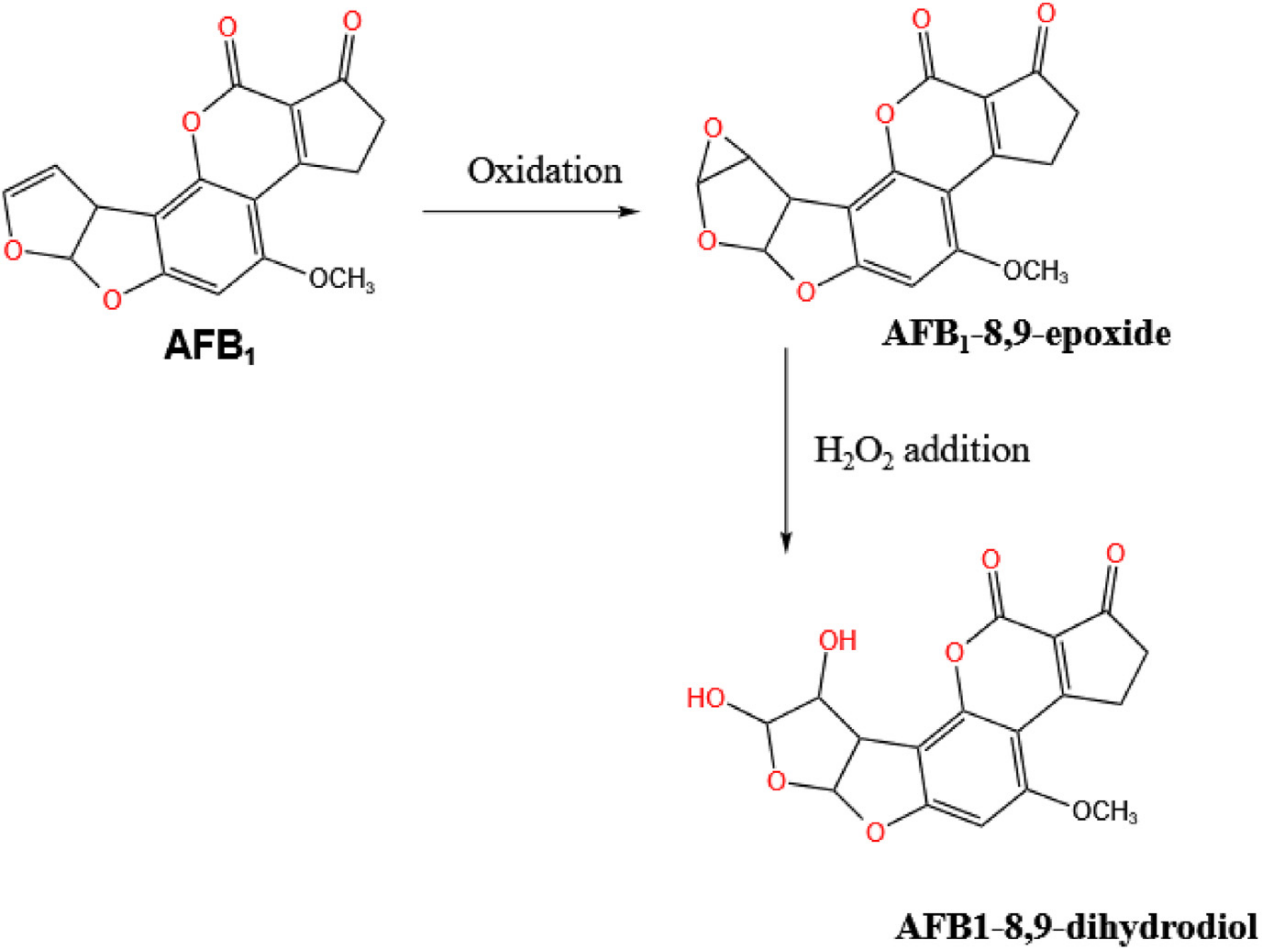
The epoxidation degradation pathway of AFB1. The furan ring in AFB1 undergoes epoxidation and further addition reaction to produce AFB1-8,9-dihydrodiol. AFB1 = aflatoxin B1.

AFB1 contains a dihydrofuran structure which is prone to epoxidation. The epoxide thus formed can readily combine with nucleic acid and proteins and hence have a toxic effect. Certain aflatoxin-degrading enzymes can react with this part of the AFB1 molecule to form AFB1-epoxide which is further converted into AFB1-dihydrodiol to achieve detoxification (Fig. 4).

Alberts et al. (2009) first explored the use of fungus-derived laccases to degrade AFB1 in 2002. Using different media, it was found that pure fungus *Coriolus versicolor* laccase can change the double bond in the furan ring of the AFB1 molecule, thus affecting its fluorescent properties and mutagenicity. Other enzymes have subsequently been shown to have the ability to cleave furan rings. For example, Cao et al. (2011) purified aflatoxin oxidase (AFO) from *Armillariella tabescens* (strain E-20) and found that it is capable of cracking the furan ring in AFB1 thus causing a change in molecular polarity. Wang et al. (2011) found that manganese peroxidase from white rot fungus *Phanerochaete sordida* YK-624 not only leads to the oxidation of AFB1 to form AFB1-8, 9-epoxide but also degrades it by creating two hydroxyl groups to form the final product AFB1-8, 9-dihydrodiol (Fig. 4). The toxicity and mutagenic activity of the aflatoxin are thus significantly reduced and its degradation rate is up to 86.0%.

Wu et al. (2015) further demonstrated the cleavage of the furan ring in AFB1 at the 8, 9-dihydrofuran double bond using AFO isolated from *Pichia pastoris* GS115 PSA. Their results show that the action of the AFO is oxygen-dependent and produces hydrogen peroxide which may play an important role in the detoxification process. In addition, the low *K*_m_ value for the AFO–AFB1 reaction (0.33 μmol/l) indicates that the AFO is highly selective towards AFB1.

Liu et al. (2021) proposed that recombinant laccase Lac3 may cleave AFB1 into small molecular polypeptides and amino acids that contain functional groups such as H^+^ and –NH_2_. New degradation products, such as C_16_H_22_O_4_, C_14_H_16_N_2_O_2_, C_7_H_12_N_6_O, and C_24_H_30_O_6_, were thus produced by addition, substitution, or oxidation reactions at the toxic site in the AFB1 molecule. Two possible degradation pathways were proposed: (1) AFB1 continuously loses –CO and then reacts with H_2_O, H^+^, and –NH_2_ to break the double bond in the furan ring; (2) decarbonylation occurs in the AFB1 after the loss of –CO and the further reaction with H^+^ destroys the double bond.

**Fig. 5:**
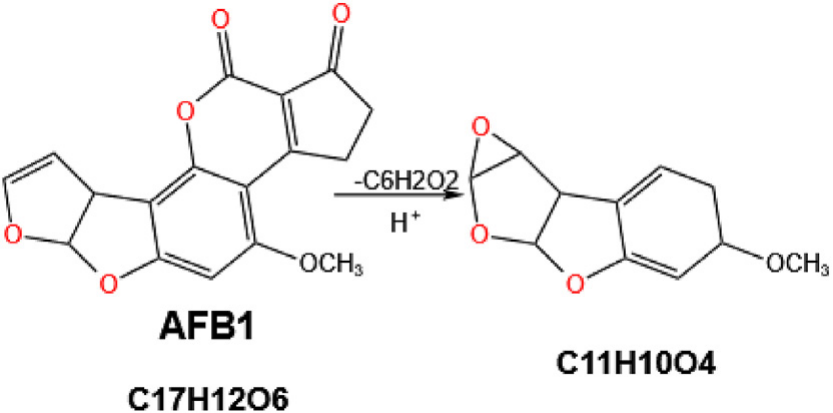
The cleavage of lactone ring and furan ring in AFB1. After completing the cleavage of the lactone ring, the enzyme can continue to act on the furan ring to produce epoxides, which then further participate in the addition reaction. AFB1 = aflatoxin B1.

As the lactone ring is extremely unstable, epoxidation of the double bond in the terminal furan ring in AFB1 can also lead to the rupture of the lactone ring system at the same time, resulting in the formation of C_11_H_10_O_4_, as illustrated in Fig. 5 (Zhang et al., 2021).

#### 2.2.3 Miscellaneous reactions

In addition to the two key sites mentioned above, there are other degradation modes of AFB1 that involve hydroxylation, reduction, and demethylation. Taylor et al. (2010) found that F_420_H_2_ reductases can catalyze the reduction of the double bonds in the α-β-unsaturated ester parts of aflatoxins thus activating the molecules for spontaneous hydrolysis and detoxification. Guo et al. (2020) discovered that the protein CotA produced from *Bacillus licheniformis* ANSB821 by allogenic expression could not only degrade AFB1 in the absence of a redox mediator but also degrade it into AFB1 C3-hydroxyl derivatives (AFQ1 and epi-AFQ1) by C3 hydroxylation. Furthermore, clinical trials found that the two derivatives had little effect on the activity of human hepatocytes (L-02).

Nakazato et al. (1990) found that intracellular enzymes from *Eurotium herbariorum* can convert AFB1 to aflatoxicol (AFL-A stereoisomer) by reducing the cyclopentenone carbonyl group in AFB1. The AFL-A could then be converted into the other stereoisomer (AFL-B) by lowering the pH of the medium. The enzyme CYP321A1 derived from the corn earworm (*Helicoverpa zea*) has similarly been found to convert AFB1 into the less toxic AFP1 through a process of demethylation (Niu et al., 2008).

In addition to the above degradation pathways, recent studies have found that aflatoxins can interact near the T1 copper center in laccase via hydrogen bonding and hydrophobic interactions with amino acid residues (Liu et al., 2020). Xiong et al. (2022) have recently found that Lys-153, Arg-268, and His-87 residues in laccase might play an important role in the binding of aflatoxin to the laccase. In addition, Zhou et al. (2022) also measured the binding affinity of hydrogen bonds and amino acid residues (Asn336, Asp207, Val391, and Thr165) in laccase with AFB1, and speculated that mutations in these residues may increase their affinity for AFB1.

### 2.3 Application of AFB1-degrading enzymes in animal production

With the continuous research of biological detoxification in recent years, it is found that the usage of biological enzymes to degrade mycotoxins is very beneficial. They can directly act on the toxic structure of mycotoxins in contaminated diets and effectively transform them into low-toxic or non-toxic products, which will be of great significance for the safety of feed foods. In addition, with the continuous exploration of AFB1-degrading enzymes, it is found that they may also be developed for reducing the toxicity of animal mycotoxins, improving animal production performance, enhancing antioxidant enzyme activity, and protecting the body from mycotoxins damage as feed additives.

Fan et al. (2013) found that the addition of *B. subtilis* ANSB060 to broiler diets can offset the negative effects of AFB1 on ADG (average daily gain), ADFI (average daily feed intake) and meat quality of broilers, while also reducing the residual amount of aflatoxins in the liver. In this regard, Zhang et al. (2016) also found that this bacterium has the same improvement effect on the production performance of Cherry Valley ducks. In addition, Ma et al. (2012) found that adding *B. subtilis* ANSB060 can also improve the strength of the egg shell. Based on the cell-free culture supernatant of *B. subtilis* ANSB060 having a good degradative effect on aflatoxin (Gao et al., 2011), Fan et al. (2013) pointed out that the bacteria may detoxify through enzymatic actions rather than binding or absorption of aflatoxin into the cell wall. Therefore, in the above experiments, perhaps the real protective effect on animal performance is attributed to the presence of aflatoxins-degrading enzymes.

Liu et al. (2021) found that laccase can protect animals from damage by alleviating AFB1-induced oxidative stress and inflammation, decreasing liver cell apoptosis, and reducing pathological damage to liver and kidney tissues. In addition, biological enzymes can also reduce the body damage caused by AFB1. For example, Li et al. (2019) found that thioredoxin (Trx) combined with MSMEG_5998, a F_420_H_2_ dependent reductase (FDR) produced by *Mycobacterium smegmatis*, can reduce AFB1-induced cytotoxicity of HepG2 cells by improving DNA damage and p53-mediated apoptosis, and the Trx link also enhances the enzyme activity, which is even more protective than natural MSMEG_5998. Similarly, the enzymes in the cell-free culture supernatant of *B. subtilis* ANSB060 have a protective effect on the pathological changes and antioxidant enzyme activity of liver tissue in broilers poisoned by aflatoxins (Fan et al., 2015). Murcia and Diaz (2021) found that glutathione S-transferase enzyme activity can respond to a wide range of AFB1 concentrations in chickens. Moreover, the nucleophilic capture of aflatoxin B1-8,9-epoxide (AFBO) by glutathione S-transferase enzyme is the main way to inactivate AFBO. This may explain the strong resistance of chickens to the carcinogenic effects of AFB1. Thus, AFB1-degrading enzyme may play an important role in animal production, but more experiments in vivo are needed to prove it.

Recently, a new application combining physical and biological detoxification, for example, a compound mycotoxin antidote (CMD) is formed by aflatoxin-degrading enzyme (ADE), montmorillonite and other physical adsorbents, and compound probiotics, which is used as feed additive imparting beneficial effects on animal production. They can significantly alleviate the negative effects of AFB1 on the broiler production performance and nutrient metabolism rate by improving ADG, ADFI, and feed conversion rate of broiler chickens (Guo et al., 2021, 2023; Zuo et al., 2013). In addition, adding CMD significantly improved the inflammatory response. The possible mechanisms are: (1) The degradation of AFB1 by CMD reduces the absorption and residue of broilers, reducing inflammation and tissue damage; (2) CMD alleviates inflammatory response by decreasing the expression of inflammatory cytokines; and (3) the addition of CMD can maintain the stability of intestinal microbiota, change the enrichment of kinases related to any inflammatory pathway, and reduce the toxicity of AFB1 to broilers (Guo et al., 2022).

In conclusion, the practical application of AFB1-degrading enzymes warrants further exploration, which provides hope for their future application in animals.

## 3 Zearalenone

**Fig. 6:**
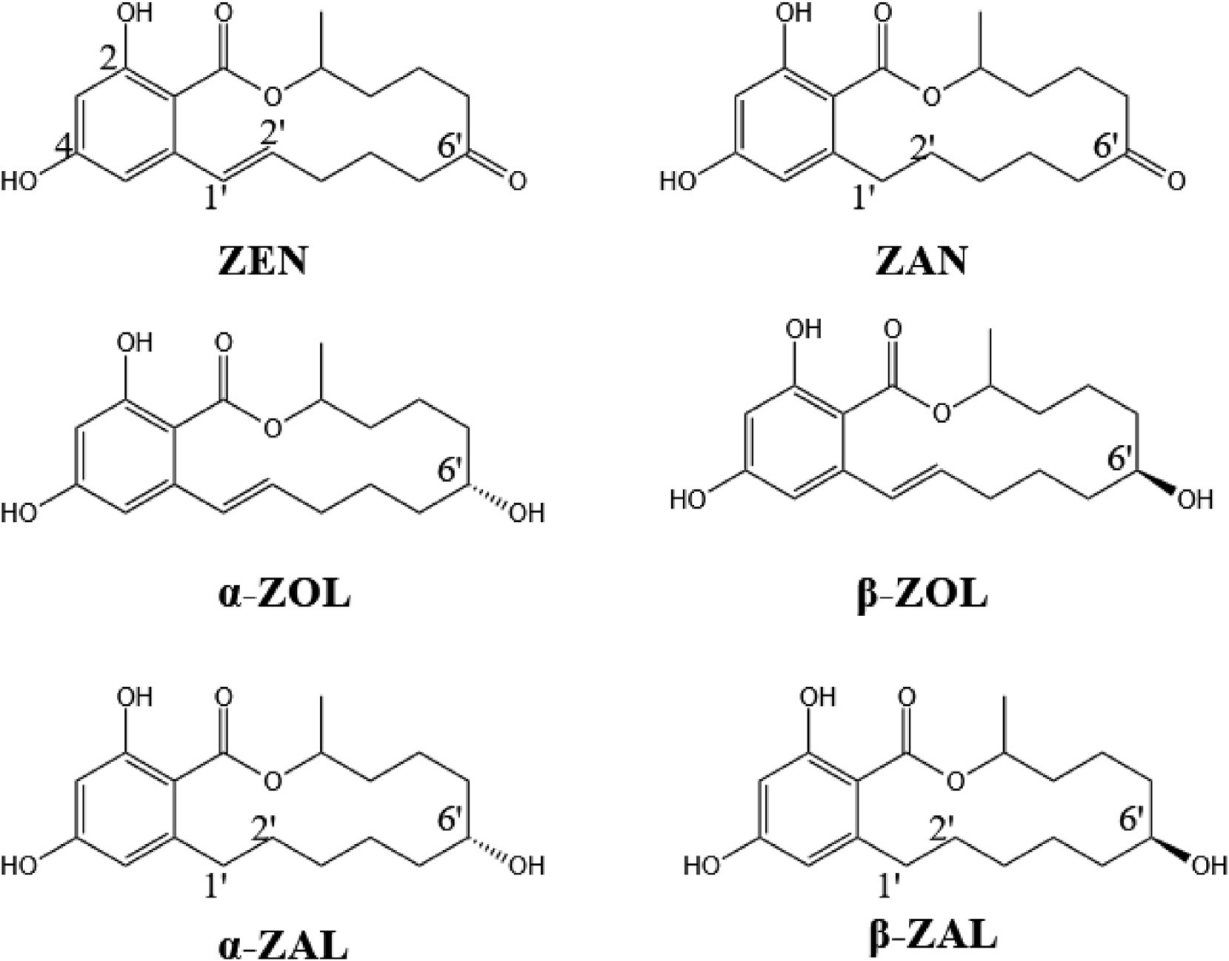
Molecular structures of ZEN and its derivatives. Among them, ZEN and ZAN are different at the 1′-2′-positions, ZOL is different from ZEN at the 6′-position, and ZAL is a product of changes in the 1′-2′-positions of ZOL; α and β represent cis and trans structures, respectively. ZEN = zearalenone; ZAN = zearalanone; ZOL = zearalenol; ZAL = zearalanol.

ZEN is a mycotoxin that is mainly produced by *Fusarium* fungi and is often found in various grains such as maize and wheat. As a result, it can seriously affect the yields of these crops (Jing et al., 2022). ZEN has a resorcylic acid lactone structure and various derivatives can be produced by making changes to its structure (generally via functional differences at the C1 and C6 carbons in its lactone ring structure). Common derivatives include α/β-zearalenol (α/β-ZOL), α/β-zearalanol (α/β-ZAL), and zearalanone (ZAN), as shown in Fig. 6. Of these, α-ZOL and α-ZAL are more toxic than ZEN (Ballo et al., 2023).

As the structure of ZEN is similar to that of natural estrogen, it can bind to estrogen receptors in the body. It thus acts as nonsteroidal estrogen, disrupting reproductive hormones and testicular development systems regulated by hormone-related genes. ZEN can also induce genotoxicity, hepatotoxicity, immunotoxicity, and cytotoxicity through oxidative damage, endoplasmic reticulum stress, mitochondrial apoptosis, autophagy, and other pathways (Feng et al., 2022; X. Gao et al., 2022; Y. Li et al., 2021; Zhu et al., 2021).

### 3.1 Enzymatic degradation of ZEN

There are three types of enzymes that can degrade ZEN, namely: laccase, lactase, and peroxidase. Table 2 presents the ZEN-degrading enzymes from different microbial sources which have been found in the past five years. These findings outlined above provide new ideas and development space for the enzymatic detoxification of ZEN.

**Table 2:** ZEN-degrading enzymes from different microbial sources.

Enzyme abbreviations	Sources	Enzyme names	Identified substrates	Degradation conditions and effects	References
*Bs*DyP	*Bacillus subtilis* SCK6	Peroxidase	ZEN	The degradation efficiency was 84.65% at pH 4.0 and 30 °C for 48 h.	Qin et al. (2021a)
*St*MCO	*Streptomyces thermocarboxydus*	Multicopper oxidases	ZEN	At pH 7.0 and 30 °C for 24 h, at 1 mmol/L ABTS, the degradation efficiency was 100%.	Qin et al. (2021c)
				At pH 7.0 and 30 °C for 24 h, at 1 mmol/L AS, the degradation efficiency was 97.35%.	
				At pH 7.0 and 30 °C for 24 h, at 1 mmol/L ferulic acid, the degradation efficiency was 70.05%.	
ZHD-LD	*Exophiala spinifera*	Lactonase	ZEN	The optimal activity conditions are 50 °C and pH 9.0.	Zhang et al. (2022)
ZENG	*Gliocladium roseum*	Lactonase	ZEN	The degradation efficiency was 70% at pH 7.0 and 38 °C for 3 h.	Zhang et al. (2020)
			α-ZOL	The degradation efficiency was 55% at pH 7.0 and 38 °C for 3 h.	
Lac2	*Pleurotus pulmonarius*	Laccase	ZEN	At pH 7.0, and 37 °C for 1 h, at 0.5 mmol/L AS, the degradation efficiency reached 99.82%.	Song et al. (2021)
*Bs*CotA	*Bacillus subtilis*	Laccase	ZEN	At pH 7.0 and 30 °C for 10 h, at 1 mmol/L methyl syringate, the degradation efficiency was 100%.	Wang et al. (2019)
*St*DyP	*Streptomyces thermocarboxydus* 41291	Peroxidase	ZEN	At pH 5.0 and 30 °C for 12 h, at 1 mmol/L MnSO_4_ and 0.1 mmol/L H_2_O_2_, the degradation efficiency was 45%.	Qin et al. (2021b)
				At pH 5.0 and 30 °C for 6 h, at 1 mmol/L 1-HBT and 0.1 mmol/L H_2_O_2_, the degradation efficiency was 98.76%.	
FSZ	*Aspergillus niger* ZEN-S-FS10	NR^1^	ZEN	The degradation efficiency was 75% at pH 7.0 and 28 °C for 24 h.	Ji et al. (2022)
			α-ZAL	The degradation efficiency was 25% at pH 7.0 and 28 °C for 24 h.	
			β-ZOL	The degradation efficiency was 50% at pH 7.0 and 28 °C for 24 h.	
			ZAN	The degradation efficiency was 80% at pH 7.0 and 28 °C for 24 h.	
ZenH	*Aeromicrobium* strain	Lactone hydrolase	ZEN	The degradation efficiency of FCC was 75.7% at pH 7.0 and 30 °C for 30 min.	Hu et al. (2023)
				The degradation efficiency of EAZ was 85.3% at pH 7.0 and 30 °C for 30 min.	
Zhd518	NR^1^	Lactone hydrolase	ZEN	The optimal activity conditions are 40 °C and pH 8.0.	Wang et al. (2018)
ZHD607	*Phialophora americana*	Lactone hydrolase	ZEN	The optimal activity conditions are 35 °C and pH 8.0.	Yu et al. (2020)
PR-ZHD	*Clonostachys rosea* strain GRZ7	Lactone hydrolase	ZEN	The degradation efficiency was 100% at pH 8.0 and 20 °C for 3 h.	Shcherbakova et al. (2020)
ZHD-P	*Trichoderma aggressivum*	Lactone hydrolase	ZEN	The optimal temperature of 45 °C and pH 7.5–9.0.	Chen et al. (2021)

ZEN = zearalenone; ZAN = zearalanone; ZOL = zearalenol; ZAL = zearalanol; AS = acetosyringone; ABTS = 2,2′-Azinobis-(3-ethylbenzthiazoline-6-sulphonate); 1-HBT = 1-hydroxybenzotriazole; MnSO_4_ = manganese sulfate; FCC = naturally-contaminated corn samples; EAZ = exogenously ZEN-contaminated corn samples.

1 NR denotes information that has not been reported.

### 3.2 Action mechanisms of ZEN-degrading enzymes

**Fig. 7:**
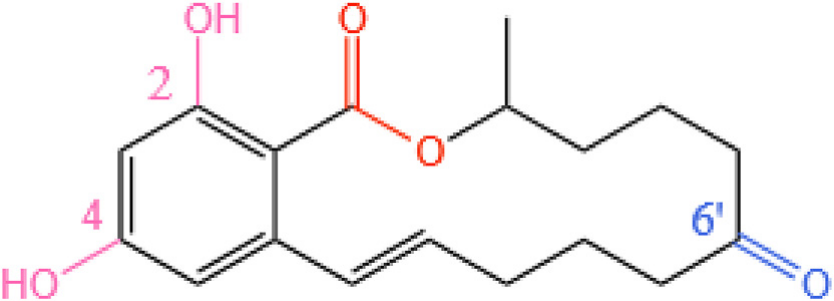
The chemical structure of ZEN highlighting the functional groups that can be targeted for degradation purposes. The hydroxyl groups are colored pink, the lactone bond is colored orange, and the ketone carbonyl group is colored blue. ZEN = zearalenone.

ZEN can be degraded via several pathways which can involve hydrolysis, reduction, glycosylation, and other processes. At present, the main mechanism involved in enzyme-induced ZEN degradation is to destroy the lactone structure of ZEN using lactonase or laccase (possibly supplemented with the oxidation/modification of the C6′ ketone group and C2/C4 hydroxyl groups shown in Fig. 7).

#### 3.2.1 Changing the toxic lactone ring structure in ZEN

Currently, the most studied ZEN-degrading enzyme is lactase whose encoding gene is *zdh101*. At present, a large number of experiments have cloned a variety of recombinant ZHD101 enzymes and no obvious side effects on cell growth, acid resistance, and bile salt tolerance were observed in the production process (Higa-Nishiyama et al., 2005; Yang et al., 2017).

The lactone ring structure present in ZEN may be the fundamental toxic structure as various ZEN metabolites with macrocyclic lactone ring structures (α/β-ZAL, α/β-ZEL) show significant toxicity. The lactone ring in ZEN can be readily hydrolyzed using esterase or acids/bases. After the lactone ring structure is ruptured, the ring opens up to form a straight chain structure which cannot combine with estrogen receptors (thus achieving detoxification). Xu et al. (2020) screened and identified a key gene (BAMF_RS30125) from *Bacillus amyloliquefaciens* H6 that is capable of degrading ZEN. After heterologous expression, its protein sequence was analyzed and it was found that BAMF_RS30125 is a protease belonging to the YBGC/FADM family of CoA thioesterases. It can open the lactone ring in ZEN by destroying the lactone bond and has been named ZTE138.

**Fig. 8:**
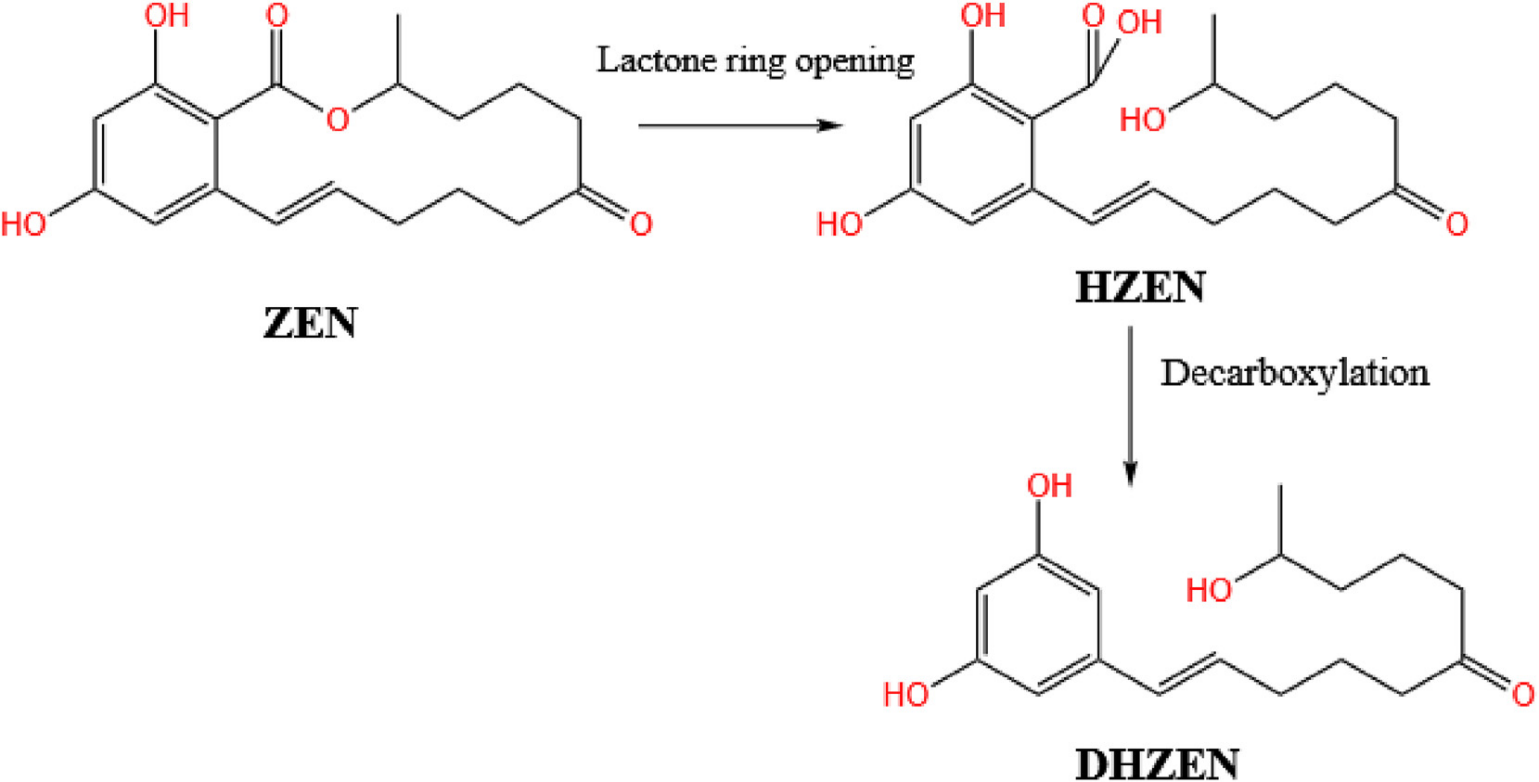
The biotransformation of ZEN. In this case, the lactone ring in ZEN is hydrolyzed to form the products without estrogenic toxicity called HZEN and DHZEN. ZEN = zearalenone; HZEN = hydrolyzed zearalenone; DHZEN = decarboxylated hydrolyzed zearalenone.

The enzyme encoded by *zhd101* has been reported to degrade ZEN into a non-estrogenic compound whose formal name is 1-(3, 5-dihydroxyphenyl)-10-hydroxyundec-1-en-6-one (Takahashi-Ando et al., 2002). While in experiments by Fruhauf et al. (2019), the two metabolites produced in these two processes were named as hydrolyzed zearalenone (HZEN) and decarboxylated hydrolyzed zearalenone (DHZEN), as shown in Fig. 8. Gruber-Dorninger et al. (2021) used hydrolase ZenA (ZEN*zyme*, BIOMIN Holding GmbH, Getzersdorf, Austria) as a feed additive and found that the enzyme not only promoted the degradation of ZEN to non-estrogenic metabolites HZEN and DHZEN, but also prevented the formation of α-ZEL in dairy rumen fluid. In addition, ZenA plays the same role in the gastrointestinal tract of pigs (Gruber-Dorninger et al., 2023).The degradation of ZEN by *Bacillus* culture extracts has also been found to be accompanied by CO_2_ generation (Tinyiro et al., 2011). This indicates that decarboxylation is taking place and results in less toxic or even nontoxic products.

Zearalenone hydrolase (ZHD) has been shown to be an α/β-hydrolase that degrades ZEN and its derivatives by cracking open their lactone rings. Qi et al. (2017) proposed two different catalytic mechanisms for the hydrolysis process. The first is a nucleophilic mechanism. It usually begins with the serine moiety attacking the carbonyl carbon atom in the substrate to form the signature tetrahedral intermediate. This nucleophilic mechanism has recently been used to elucidate the hydrolysis of *N*-acyl homoserine lactase AidH. The second hydrolysis mechanism is often referred to as the ‘general alkali mechanism’. In the case of ZHD, the structure of the enzyme–product complex provides valuable information on the final stage of the catalysis process.

#### 3.2.2 Changing the toxic ketone structure in ZEN

**Fig. 9:**
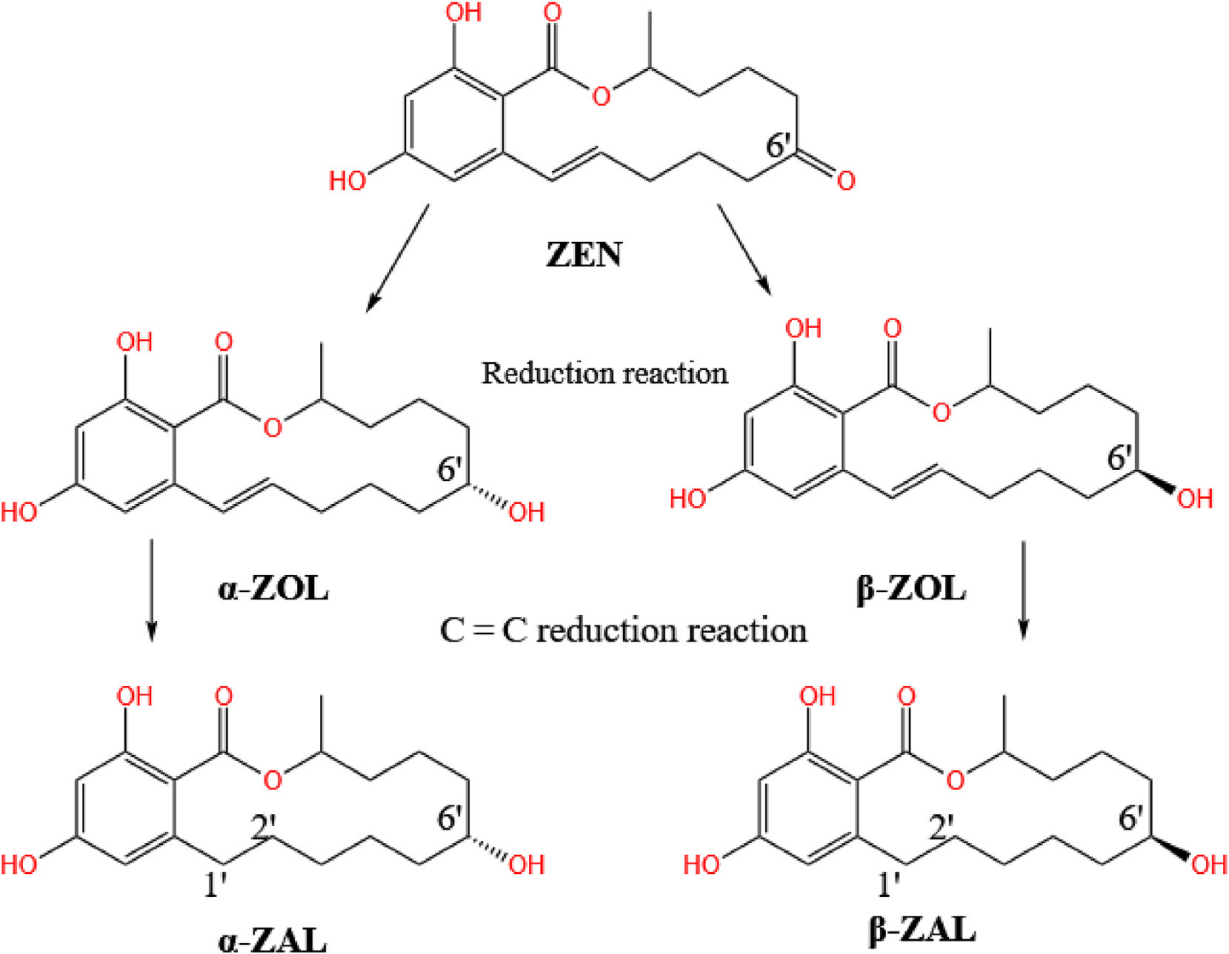
The transformation pathway from ZEN to its derivatives. ZEN can be converted into its derivatives through multiple reduction reaction. ZEN = zearalenone.

The C6′-ketocarbonyl group in ZEN is easily reduced. The addition of hydrogen atoms directly forms zearalenol (α/β-ZOL). Further hydrogenation of the C1′ = C2′ double bond leads to the formation of the corresponding zearalanol (α/β-ZAL), as shown in Fig. 9 (Pompa et al., 1988). The toxicity with estrogenic effect via α-ZOL is significantly higher than that of ZEN, while β-ZOL is less toxic than ZEN. Due to the combined estrogenicity of the generated products, conversion to ZOL is therefore considered to be an ineffective detoxification route (Borzekowski et al., 2019).

**Fig. 10:**
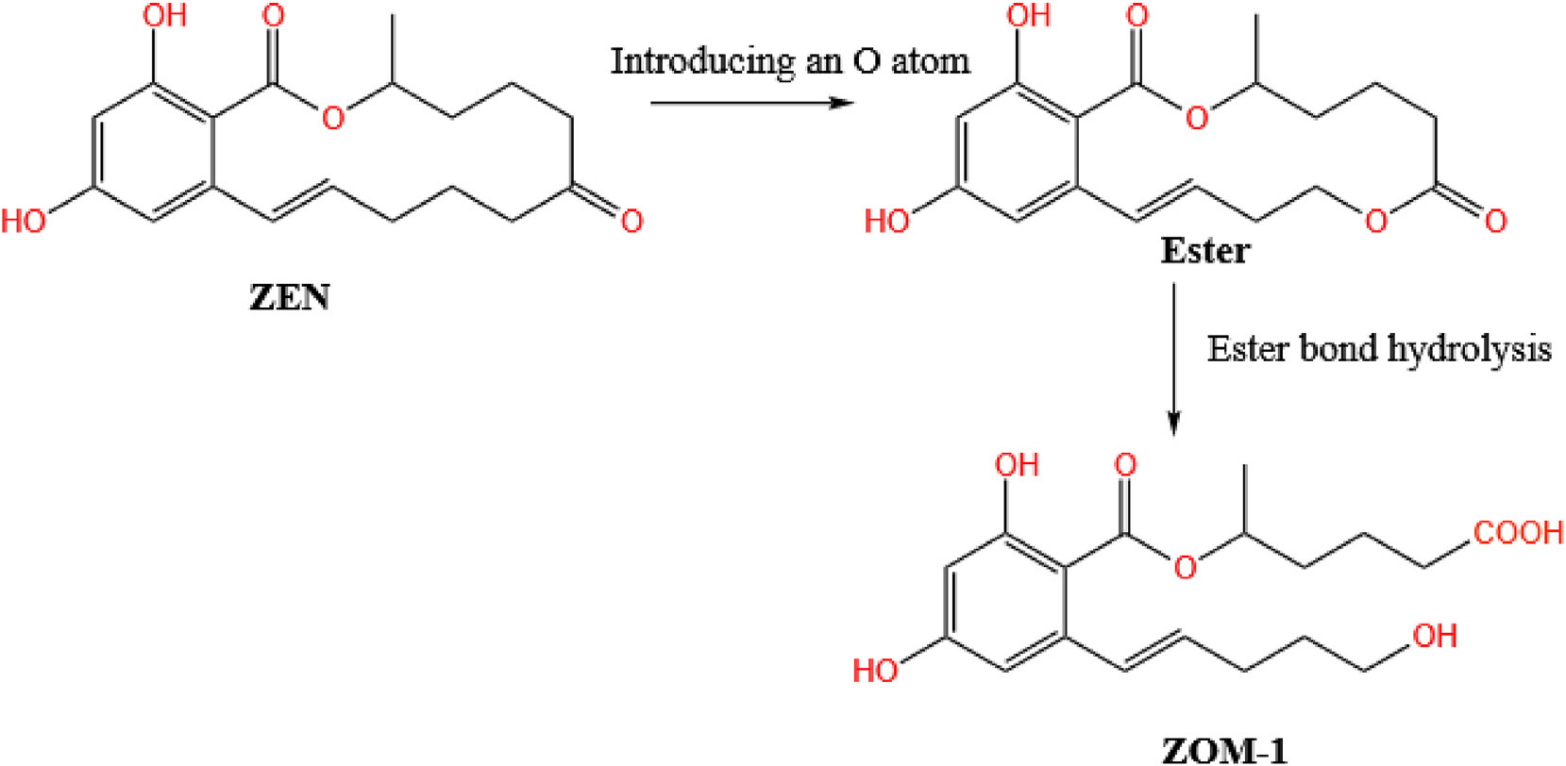
Another route available for the biotransformation of ZEN. Here, the ketonic carbonyl group is cleaved to generate a degradation product containing carboxyl and hydroxyl groups called ZOM-1 (C_18_H_24_O_7_). ZEN = zearalenone; ZOM = ZEN-derived metabolite.

The C6′-ketocarbonyl group in ZEN can also be converted into an ester group by introducing an oxygen atom into the ortho site. This ester group can then be hydrolyzed to produce a degradation product containing carboxyl and hydroxyl groups called ZOM-1. The ring-opening and formation of carboxyl and hydroxyl groups at the ketone group in the ZEN macro-ring is different from the hydrolysis and decarboxylation of the pre-existing lactone group in ZEN. Vekiru et al. (2010) confirmed that ZOM-1 is formed. This new metabolite of ZEN was identified using nuclear magnetic resonance and has the structure as shown in Fig. 10. Its formal name is (5S)-5-({2,4-dihydroxy-6-[(1E)-5-hydroxypent-1-en-1-yl]benzoyl}oxy)hexanoic acid (molecular formula: C_18_H_24_O_7_). The first of these reactions usually can require a nicotinamide adenine dinucleotide phosphate (NADPH)-dependent Baeyer-Villiger monooxygenase. It has been reported that an untypical cytochrome P450 monooxygenase, and an oxidase encoding the aflatoxin biosynthetic cluster gene *AflY*, may also be involved in this reaction (Ehrlich et al., 2005; Kim et al., 2005). It is worth mentioning that ZOM-1 has no estrogenic effect in vivo and does not interact with estrogen receptor proteins in vitro (Vekiru et al., 2010).

Clearly, the identification of key genes or degradation enzymes involved in ZEN detoxification can provide valuable new insights into the detoxification pathways available for ZEN (Vekiru et al., 2010).

#### 3.2.3 Changing the toxic C2/C4 hydroxyl groups in ZEN

ZEN is a mycotoxin with the dihydroxybenzoic lactone structure. New degradation products that are less toxic may therefore be (potentially) generated by modifying the phenolic hydroxyl groups attached to the C2 and C4 atoms in ZEN. It has been reported that ZEN can be glycosylated using appropriate enzymes to produce ZEN glucosides which can reduce the acute toxicity of the ZEN towards humans and animals.

Poppenberger et al. (2006) a cloned *Arabidopsis* UDP-glucosyltransferase (UGT) that catalyzes the glycosylation of ZEN via the –OH group on the C4 carbon, thus converting it to zearalenone-4-*O*-glucoside (ZEN-4-*O*-Glc). Other scholars also effectively transformed ZEN and its metabolites (α/β-ZEL) using a recombinant barley glucosyltransferase (*Hv*UGT14077) thus generating ZEN-2-glucoside and ZEN-4-glucoside, and ZEN-2-*O*-β-lucoside and ZEN-4-*O*-β-glucoside (Kovalsky et al., 2014; Michlmayr et al., 2017). ZEN can also undergo sulfation which converts it into ZEN-sulfate (El-Sharkaway et al., 1991). However, neither glycosylation nor sulfation can be considered an effective detoxification method. This is because it has been found that these conjugates can be rapidly hydrolyzed in the gastrointestinal tract and reconverted back to the parent mycotoxin, effectively resulting in the same overall toxicity as ZEN (Binder et al., 2017; Kovalsky et al., 2014).

Regardless of whether or not the phenyl hydroxyl groups are modified, the dihydroxyl benzene ring structure cannot be destroyed. This makes it difficult to further degrade the products into smaller molecules. Therefore, cleavage of the dihydroxy benzene ring can also be an effective method of detoxification. It has been reported that extracellular enzymes in the filtrate of *Aspergillus niger* FS10 can be used to convert ZEN into other products (Sun et al., 2014). Two intermediates, ZEN-A and ZEN-B, were determined but ZEN-B was found to have no absorption peaks in the ultraviolet part of the spectrum. This indicates that the ZEN-B derivative has a fractured benzene ring. Yu et al. (2011) found that the enzymatic action of *Acinetobacter* sp. SM04 can also destroy the dihydroxy benzene ring and degrade ZEN into two compounds with absorption spectra different from that of ZEN, namely, ZEN-1 and ZEN-2. The ZEN was degraded into two carboxylic acids by the isolated peroxidase. Further tests showed that the degradation product had no estrogenic activity against MCF-7 cells (Tang et al., 2013). Therefore, this dihydroxy benzene ring-opening reaction pathway should be further developed and can be expected to lead to new ways of enhancing the biodetoxification of ZEN.

In summary, further research on the structure and toxicity of ZEN metabolites, and the degradation mechanisms by which ZEN-degrading enzymes function, can be expected to further promote the development of new and improved microbial ZEN detoxification strategies.

### 3.3 Application of ZEN-degrading enzymes in animal production

ZEN is a common contaminant in animal feed and is known for its estrogenic effects on animals. For ZEN, ZEN-degrading enzymes are a promising strategy to counteract its negative effects on animal production.

Some researchers have found that certain cultures of ZEN-degrading bacteria can protect animal from the effects of ZEN, and this protection is likely to depend on the action of the enzyme. For example, *B. subtilis* ANSB01G culture can alleviate the toxicity of ZEN on growth performance, reproductive organs and histopathological changes of sows (Zhou et al., 2020; Zhao et al., 2015). In addition, Danicke et al. (2023) found that the bacterial enzyme ZenA could hydrolyze ZEN to HZEN and DHZEN when added to ZEN-contaminated piglet diets, significantly reducing the estrogen response, and ameliorating the ZEN-induced reduction in uterine and ovarian relative weight. Gruber-Dorninger et al. (2021) also demonstrated that ZEN is converted into the highly estrogenic metabolite α-ZEL in bovine rumen fluid. After receiving ZenA as a feed additive, α-ZEL production was significantly reduced, and HZEN production and decarboxylated HZEN production increased, counteracting the estrogen effect of ZEN on cattle. Later, they found that ZenA added to feed can also reduce the concentration of ZEN in the gastrointestinal tract of three single-stomach animals (namely pigs, chickens, and rainbow trout) (Gruber-Dorninger et al., 2023).

Similarly, the experiments of Tso et al. (2019) found by the finding that enzyme degradation reagents have a higher removal rate than the adsorbent on simulated pig and poultry gastrointestinal conditions, which indicates that degrading enzymes play an important role in the removal of toxins from the gastrointestinal tract, reducing the effect of ZEN on animal performance. Besides, Wang et al. (2022) further demonstrated that ZEN-degrading enzymes can counteract ZEN-induced intestinal reproductive immune axis toxicity by regulation of intestinal microbiome-derived metabolites.

The combination of complex probiotics and mycotoxin-degrading enzymes also alleviated ZEN-induced cellular injury, necrosis and inflammation in IPEC-J2 cells by positively regulating gene expression related to intestinal cell inflammation, apoptosis, nutrient transport and absorption (Huang et al., 2019). It provides a new method for alleviating the cytotoxicity of mycotoxins, protecting the normal intestinal cell structure and animal health from attack by mycotoxins.

In conclusion, the application of ZEN-degrading enzymes as feed additives may be a promising strategy to counteract the toxic effects of ZEN.

## 4 Ochratoxin A

Ochratoxins are a family of mycotoxins mainly produced by various *Aspergillus* and *Penicillium* species. They are commonly found in wheat, corn, peanuts, and other crops (Liu et al., 2022). Members include OTA, ochratoxin B (OTB), ochratoxin C (OTC), and ochratoxin α (OTα). The most toxic and most frequently occurring ochratoxin is OTA.

**Fig. 11:**
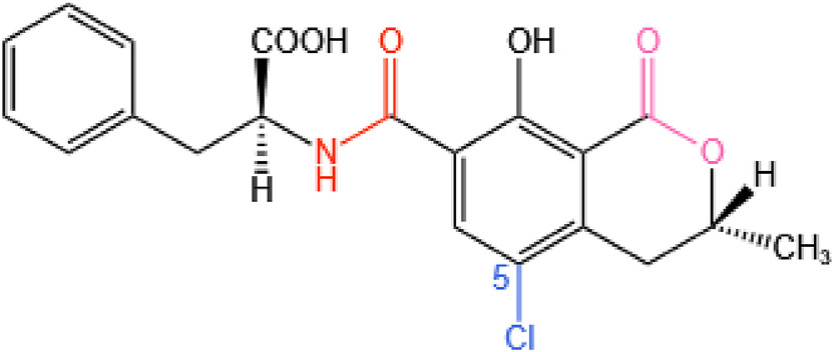
The molecular structure of OTA. The amide bond is colored orange, the lactone bond is colored pink, and chlorine atom is colored blue. OTA = ochratoxin A.

Ochratoxins have skeletons that are composed of a dihydroisocoumarin group linked to a phenylalanine group via an amide bond. OTA itself also contains a *p*-chlorophenol structure, as shown in Fig. 11 (Malir et al., 2016). OTA has immunotoxic, genotoxic, neurotoxic, and teratogenic effects (Abdelrahman et al., 2022; Park et al., 2019; Tao et al., 2018). The structure of OTA is similar to that of the essential human amino acid phenylalanine. Because of this, it can competitively inhibit the activity of phenylalanine hydroxylase in the liver and kidneys, thereby inhibiting the synthesis of the corresponding proteins (Alshannaq and Yu, 2017).

### 4.1 Enzymatic degradation of OTA

The enzymes that can be used to degrade OTA are mainly amidohydrolases, carboxypeptidases, and lipases. Table 3 presents the OTA-degrading enzymes from different microbial sources which have been found in the past five years and displays the associated degradation conditions and effects.

**Table 3:** OTA-degrading enzymes from different microbial sources.

Enzyme abbreviations	Sources	Enzyme names	Identified substrates	Degradation conditions and effects	References
POD	*Armoracia rusticana*	Commercial peroxidase	OTA	The degradation rate was 27.0% at pH 7.0 and 30 °C for 6 h.	de Oliveira et al. (2020)
*Af*OTase	*Alcaligenes faecalis* DSM 16503	Amidohydrolase	OTA	The optimal activity conditions are 50 °C and pH 6.5.	Zhang et al. (2019)
CP4	*Lysobacter* sp.CW239	Carboxypeptidase	OTA	The degradation efficiency was 36.8% at pH 7.0 and 30 °C for 24 h.	Wei et al. (2020)
DacA	*Bacillus subtilis* CW14	Carboxypeptidase	OTA	The degradation efficiency was 71.3% at pH 7.0 and 37 °C for 24 h.	Xu et al. (2021)
ADH3	*Stenotrophomonas acidaminiphila* CW117	Amidohydrolase	OTA	The degradation efficiency reached 99% at pH 8.0 and 50 °C for 90 s.	Luo et al. (2022)
Nh-9	*Bacillus velezensis* IS-6	Nudix hydrolase	OTA	The degradation efficiency was 68% at pH 7.0 and 37 °C for 24 h.	Jahan et al. (2023)
Chr1_3858681_3267	*Stenotrophomonas* sp. 043-1a	Amidohydrolase	OTA	At pH 7.5 and 37 °C for 3 h 15 min, with a dilution of 1:7 in the activity assay, the degradation efficiency was 100%.	Gonaus et al. (2023)

OTA = ochratoxin A.

### 4.2 Action mechanisms of OTA-degrading enzymes

The different degradation modes can be classified according to the reactions occurring and include hydrolysis, hydroxylation, glycation, and esterification (the hydrolysis reaction can be conducted by hydrolyzing either the amide or ester bonds). The kinds of degradation products generated are different depending on the degradation pathway involved.

#### 4.2.1 Changing the toxic amide bond in OTA

**Fig. 12:**
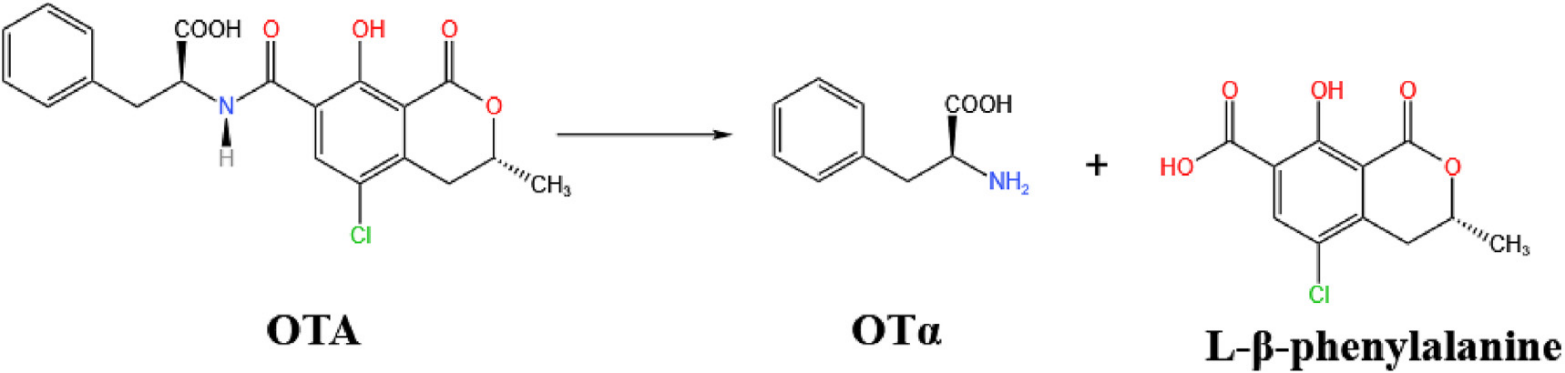
A biotransformation pathway for OTA. Here, the amide bond in OTA is hydrolyzed and cleaved to generate OTα and L-β-phenylalanine. OTA = ochratoxin A; OTα = ochratoxin α.

OTA can be biodegraded by hydrolyzing the amide bond that connects the L-β-phenylalanine molecule to the OTα part of the molecule (Fig. 12). Crude lipase products from *A. niger* have been reported to hydrolyze OTA via its amide bond (Abrunhosa and Venancio, 2007; Stander et al., 2000). Moreover, their ability to degrade OTA was found to be higher than that of CPA at pH 7.5 and 37 °C. Chang et al. (2015) isolated *B. amyloliquefaciens* ASAG1 which can effectively degrade OTA and used cloning to obtain a hydrolase (carboxypeptidase) from it. The carboxypeptidase was found to more efficiently degrade OTA in vitro, finding that it is the species most likely to be responsible for the enzymatic transformation of the OTA. Other scholars further demonstrated that after OTA reacts with peptidase, the amide bond is hydrolyzed and the OTA is cleaved into OTα and L-β-phenylalanine, as shown in Fig. 12 (Cho et al., 2016; Zhang et al., 2017). The same degradation pathway was found using the new degradation enzyme *Af*OTase purified from *Alcaligenes faecalis* DSM 16503 (Zhang et al., 2019).

Several commercial proteases have also been reported to hydrolyze OTA to OTα, e.g. protease A from *A. niger* and pancreatin from porcine pancreases (Abrunhosa et al., 2010). It is worth mentioning again that OTα and L-β-phenylalanine are both nontoxic. Furthermore, OTα is a major metabolite in animals and humans and an important metabolite in microbial and enzymatic systems. Therefore, the hydrolysis of the amide bond in OTA can be viewed as a safe and efficient degradation pathway.

#### 4.2.2 Changing the toxic isocoumarin ring structure in OTA

The isocoumarin ring in OTA can be hydroxylated under the action of oxidase to produce 4- and 10-hydroxyochratoxin A (the main product being the 4-hydroxy-OTA isomer) (Stormer and Pedersen, 1980). When OTA is hydroxylated in rats, the main hydroxyl product is 4*R*-4-hydroxy-OTA; in pigs, the main product is 4*S*-4-hydroxy-OTA. OTA in rat urine has also been reported to be hydroxylated to 4*R*-4-hydroxy-OTA. In addition, it is thought that cytochrome P450 may also be involved in the hydroxylation of OTA in rat liver microsomes.

**Fig. 13:**
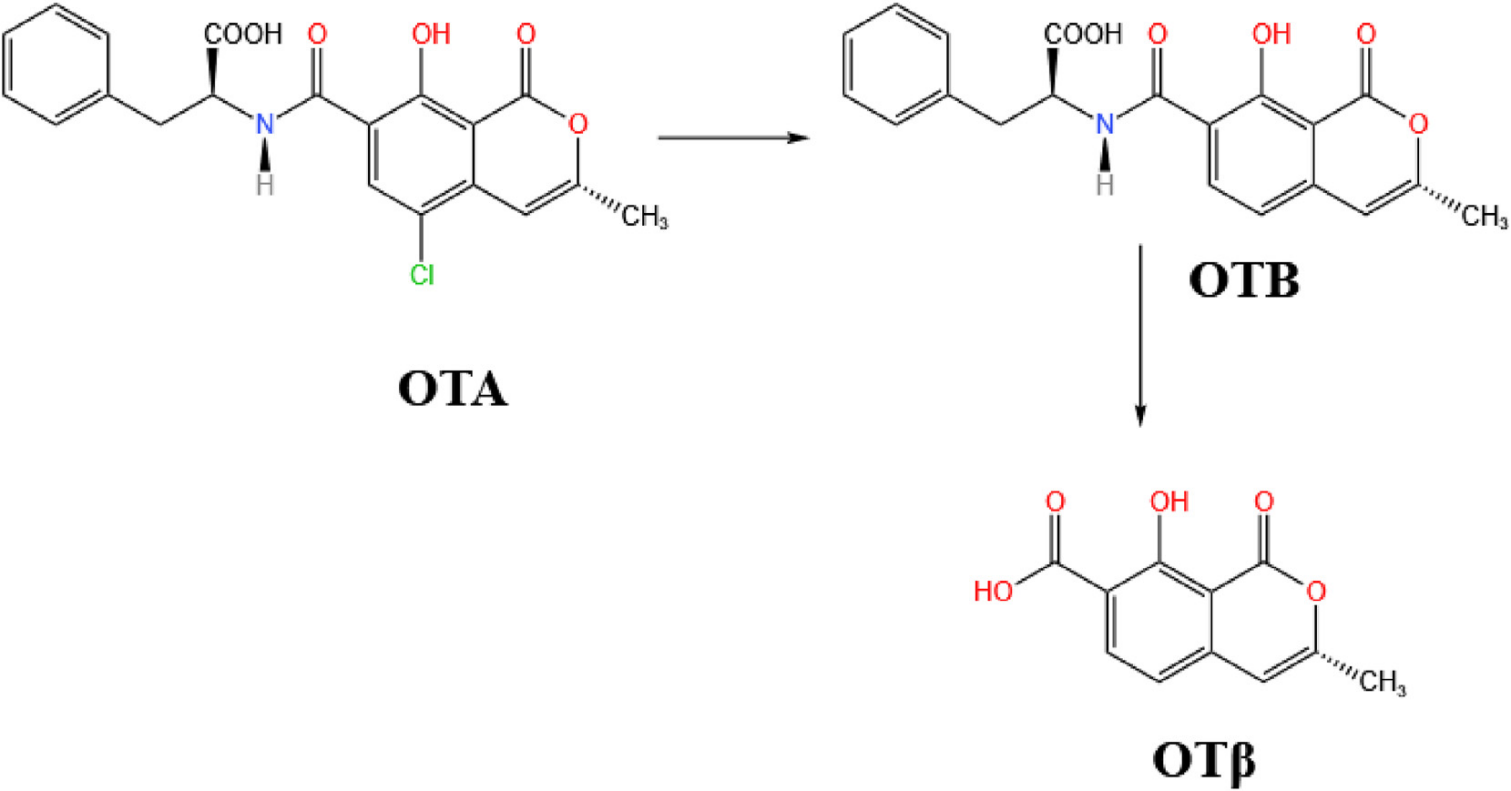
Another biotransformation pathway for OTA. In this case, the dechlorination of the OTA generates OTB which can further generate OTβ. OTA = ochratoxin A; OTB = ochratoxin B; OTβ = ochratoxin β.

The chlorine atom in the OTA structure may also contribute to its toxicity. When OTA loses the chlorine atom on the C5 atom, OTB is produced. OTB can then react further to produce other products that are less toxic, e.g. 4-hydroxy-OTB and ochratoxin β (OTβ, which is shown in Fig. 13) (El et al., 2000). OTA can also produce a quinone (OTQ)/hydroquinone (OTHQ) redox couple when it undergoes oxidative dechlorination and this may play a role in OTA-mediated genetic toxicity (Tozlovanu et al., 2006).

#### 4.2.3 Changing the lactone ring structure in OTA

Another possible degradation pathway involves the hydrolysis of the lactone ring structure in OTA, the final degradation product being the open-lactone form of OTA (OP-OTA). For example, *N*-[3-carboxy-5-chloro-2-hydroxy-4-(2-hydroxypropyl)benzoyl]-L-phenylalanine has been reported to be produced when the OTA lactone ring is opened via enzymatic hydrolysis. However, the OP-OTAs found in rodents are more toxic than the parent OTA (Li et al., 1997). This suggests that the lactone ring may not be the main structure responsible for the toxicity of OTA.

OTA can also undergo esterification and glycosylation. The glycosylation of OTA produces compounds such as hexose and pentose conjugates which can be found in animals. The formation of these kinds of more polar metabolites enables the OTA to be eliminated more rapidly (Wu et al., 2011). OTA can also be catalytically esterified to produce, for example, OTA methyl ester (in cultures of wheat and corn cells). OTA ethyl ester can also be generated via esterification. However, they are quickly hydrolyzed to OTA (by deacetylation) which may explain their similar toxicities.

In conclusion, further studies on the degradation of OTA by microbial enzymes and the toxicity and structure of its metabolites can be expected to furnish new and improved microbial OTA-detoxification strategies.

### 4.3 Application of OTA-degrading enzymes in animal production

Animal feed is often contaminated with OTA, a potent natural mycotoxin that is harmful to animal and human health and accumulates in the blood and tissues.

Elhady et al. (2022) found that the use of *B. subtilis* fermentation extract conferred a significant improvement on immune toxicity and nephrotoxicity in broilers. This protective effect can be attributed to the CP and other proteases in the fermentation extract of *B. subtilis*, which can hydrolyze OTA into non-toxic OTα. Prasad et al. (2023) found an OTA amide hydrolase, and found that OTA amide hydrolase supplementation could significantly reduce the cumulative level of OTA in pig plasma, kidney, liver, and muscle, and weaken the harmful effects of OTA on pig productivity and welfare. Therefore, the use of enzymes as feed additives may be the most promising method to reduce the harmful effects of OTA on animal production performance and improve the safety of animal food.

However, further research in vivo is needed to explore the protective ability and potential mechanisms of OTA-degrading enzymes on animal production.

## 5 Deoxynivalenol

DON, also known as vomitoxin, is a trichothecene produced by *Fusarium* species (Riahi et al., 2021). It is mainly found in grains such as wheat, barley, and corn. Intake of low doses of DON can cause damage to the intestinal barriers and immune systems of humans and animals; high doses can cause severe diarrhea, vomiting, gastrointestinal inflammation, and immunosuppression (Pinto et al., 2022). DON can also cause cytotoxicity by inhibiting the related signaling pathways, inducing oxidative stress, autophagy, apoptosis, and other pathways (Gu et al., 2019; Xiao et al., 2022).

### 5.1 Enzymatic degradation of DON

Several enzymes have been reported to transform DON into substances with low or no toxicity by destroying specific structures in the DON molecule. Table 4 presents the DON-degrading enzymes from different microbial sources which have been found in the past five years.

**Table 4:** DON-degrading enzymes from different microbial sources.

Enzyme abbreviations	Sources	Enzyme names	Identified substrates	Degradation conditions and effects	References
DDH	*Pelagibacterium alotolerans* ANSP101	Dehydrogenase	DON	The optimal activity conditions are 40 °C and pH 8.0.	Qin et al. (2022)
*Bs*DyP	*Bacillus subtilis* SCK6	Peroxidase	DON	At pH 4.0 and 30 °C for 48 h, at 1 mmol/L MnSO_4_ and 0.1 mmol/L H_2_O_2_, the degradation efficiency was 78.42%.	Qin et al. (2021a)
AKR18A1	*Sphingomonas* S3-4	Hydroxysterone reductase	DON	At NADPH, the optimal activity conditions are 55 °C and pH 9.5.	He et al. (2017)
QDDH	*Devosia mutans* D6-9	Dehydrogenase	DON	At PPQ, the optimal activity conditions are 40 °C and pH 6.0.	He et al. (2020b)
AKR13B2		Hydroxysterone reductase	3-keto-DON	At NADPH, the optimal activity conditions are 45 °C and pH 6.0.	
AKR6D1		Hydroxysterone reductase	3-keto-DON	The optimal activity conditions are 35 °C and pH 6.5.	
DepA	*Devosia mutans* 17-2-E-8	Dehydrogenase	DON	At 1 mmol/L Ca^2+^ and 0.1 mmol/L PPQ, room temperature, pH 7.5 for 12 h, the degradation degree reached 99%.	Carere et al. (2018a)
DepB	*Devosia mutans* 17-2-E-8	Hydroxysterone reductase	3-keto-DON	At 0.4 mmol/L NADPH, the optimal activity conditions are 30-35 °C and pH 7.5.	Carere et al. (2018b)

DON = deoxynivalenol; MnSO_4_ = manganese sulfate; H_2_O_2_ = hydrogen peroxide; NADPH = nicotinamide adenine dinucleotide phosphate oxidase; PPQ = pyrroloquinoline quinone.

### 5.2 Action mechanisms of DON-degrading enzymes

**Fig. 14:**
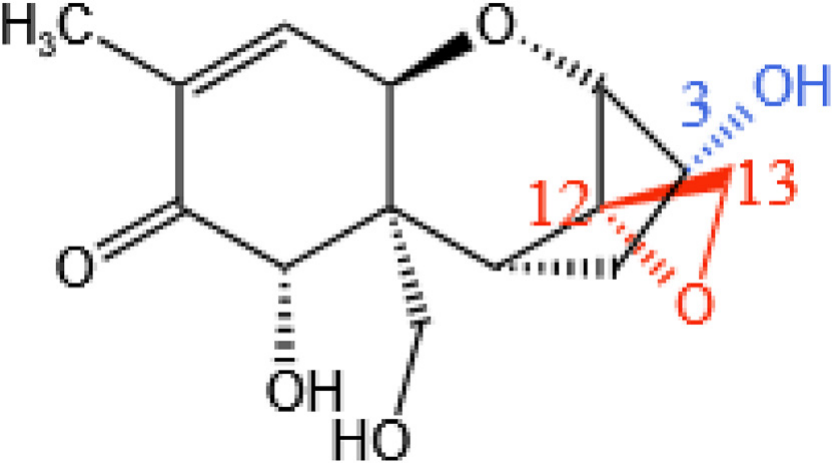
The toxic structure of DON. The C12–C13 epoxy ring is marked in orange and the C3 hydroxyl group is marked in blue. DON = deoxynivalenol.

DON is a potent inhibitor of protein synthesis. It blocks the aminoacyl site in ribosomes thus hindering aminoacyl–tRNA binding and preventing peptidyl transferase activity. The ribosome interaction is mediated by the formation of hydrogen bonds with the C12 and C13 epoxide rings and van der Waals interactions with the C3 hydroxyl group and C9 and C10 double bonds. The main toxic groups in DON are therefore the epoxy structure at the C12–C13 position and the C3 hydroxyl group, as illustrated in Fig. 14 (Yao and Long, 2020).

The degradation modes can therefore be divided into oxidation, glycosylation, de-epoxidation, and acetylation.

#### 5.2.1 Changing the toxic C12–C13 epoxy structure in DON

**Fig. 15:**
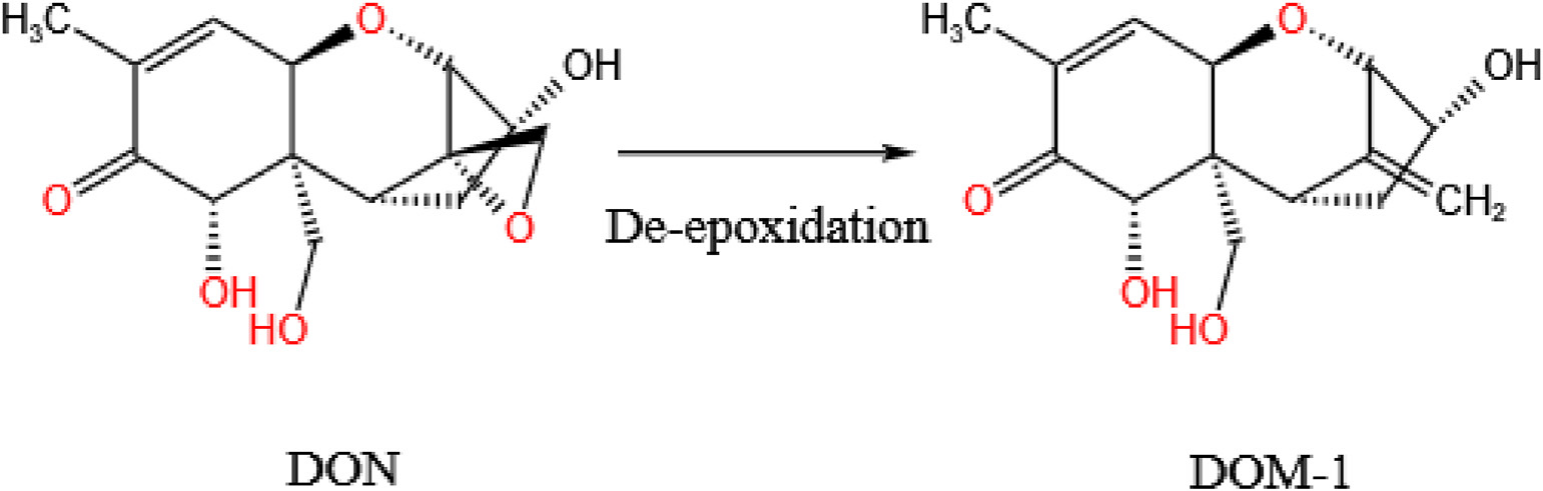
One possible biodegradation pathway available for DON. In this case, the DON undergoes de-epoxidation producing de-epoxy deoxynivalenol (DOM-1). DON = deoxynivalenol.

The toxicity of DON is largely dependent on the epoxide portion of the molecule and so opening the epoxide ring can significantly reduce its toxicity. The de-epoxidation of DON involves the removal of one oxygen atom and the introduction of three hydrogen atoms to form DOM-1, as shown in Fig. 15. At present, only a few studies have reported the degradation of DON due to enzymes acting on the C12–C13 position.

He et al. (2020a) isolated a bacterium, *Desulfitobacterium* sp. PGC-3-9, from the soil of a wheat field and found it effectively eliminated DON in wheat grains. It could also fully de-epoxidate HT-2, nivalenol, and 15-acetyl-deoxynivalenol. Moreover, the bacteria showed strong de-epoxidation activity over a wide range of pH (6–10) and temperature (15–50 °C). However, the activity of the de-epoxidase isolated from this bacterium still needs further verification. Ahad et al. (2017) obtained a highly enriched microbial community (called DX100) capable of de-epoxidating DON to form metabolites with significantly reduced toxicities. Further tests found that the cell culture lysate of this bacterial community still retained de-epoxidation activity suggesting that some cytoplasmic reductases may be responsible for the activity.

Several strains have been found that can affect the epoxy structure in DON and thus yield the metabolite DOM-1 (e.g. *Slackia* sp. D-G6 (Gao et al., 2020), *Eggerthella* sp. DII-9 (Ahad et al., 2017), *Bacillus* sp. LS100 (Li et al., 2011), and *Eubacterium* sp. BBSH 797 (Fuchs et al., 2002)). In animals, DOM-1 is generally considered to be a nontoxic metabolite (Guerrero-Netro et al., 2021; Ruhnau et al., 2021), suggesting that de-epoxidation may be a good pathway for DON degradation. However, the microbes mentioned above require an oxygen-free environment to function, which limits their practical use. This means that we ideally need aerobic bacteria that can detoxify DON. Currently, in addition to mixed cultures of microorganisms that can de-epoxidate DON to DOM-1 under aerobic conditions, there may be some facultative aerobes that may be more suitable for practical applications. Therefore, more research is required to isolate and purify new de-epoxidases from microorganisms that can degrade DON.

#### 5.2.2 Changing the toxic C3 hydroxyl group in DON

There are multiple degradation pathways available for the C3 hydroxyl group in DON. Two of the main ones are oxidation and differential isomerization of the C3 hydroxyl group which produces 3-oxo-DON (3-keto-DON) and 3-*epi*-DON, respectively. These two metabolites have been well studied in vitro and have been found to be less toxic than the maternal toxin. Two enzymes have been reported to be mainly involved in these pathways: a pyrroloquinoline quinone (PQQ)-dependent dehydrogenase and an NADPH-dependent aldo-keto reductase. Carere et al. (2018a) reported a PQQ-dependent dehydrogenase DepA derived from mutant *Devosia* spp. 17-2-E-8 which is responsible for the oxidation of the C3 hydroxyl group in DON to a keto group. On this basis, Yang et al. (2022) further revealed the interactions between dehydrogenase, cofactor, and substrate DON. It was found that the binding of DON to DepA−PQQ is likely to depend on certain unique amino acid residues. In view of this, He et al. (2020b) speculated that S497, E499, and E535 are the key residues that play a role in the DON-oxidizing activity of QDDH.

DDH has also been found to oxidize DON to 3-keto-DON in the presence of hydrogen acceptors such as phenazine methosulfate or dichlorophenolindophenol as cofactors. The two key amino acid residues in DDH that significantly affect DON degradation are serine (at position 478) and glutamic acid (at position 480) (Qin et al., 2022). In addition, AKR18A1 from the aldo-keto reductase family has been shown to oxidize DON to 3-keto-DON. The DON-degrading reaction of AKR18A1 depends on the cofactor NADPH. However, in the presence of nicotinamide adenine dinucleotide (NADH), AKR18A1 can also catalyze the reverse reaction (conversion of 3-keto-DON to DON) (He et al., 2017).

The diastereoisomer 3-*epi*-DON is produced by stereochemically converting the C3–OH center from the *S*-configuration to the *R*-configuration in a process called ‘DON differential isomerization’. Studies have shown that the 3-*epi*-DON formed after isomerization has little or no toxicity. Abraham et al. (2022) isolated a new member of the AKR18 aldo-keto reductase family, DepB_Rleg_, from *Rhizobium leguminosarum*. The enzyme was found to have the ability to utilize both NADH and NADPH as coenzymes (although the catalytic efficiency of NADH was 40 times lower than that of NADPH). They also revealed the putative roles of Lys-217, Arg-290, and Gln-294 in the specificity of the NADPH.

**Fig. 16:**
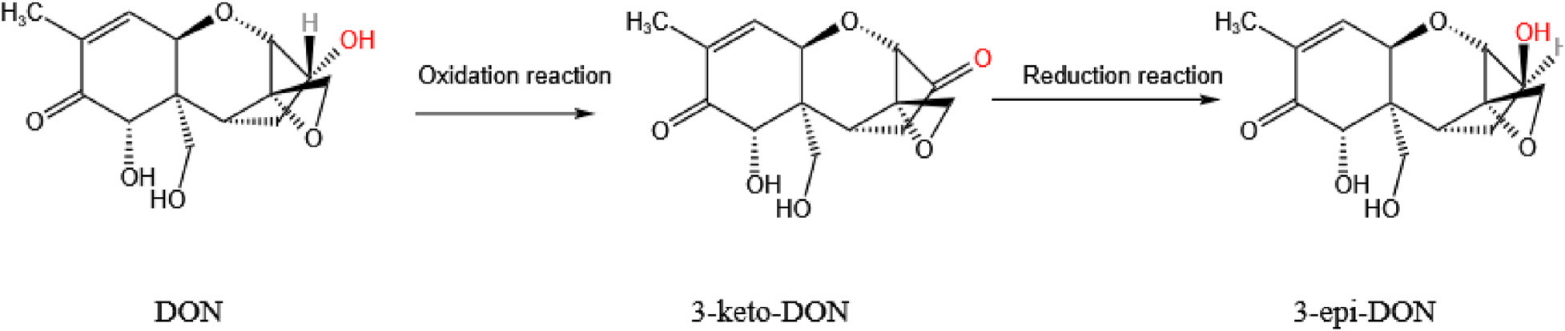
The differential isomerization of the C3 hydroxyl group in DON. DON is first oxidized to 3-keto-DON which is then reduced to 3-*epi*-DON. DON = deoxynivalenol.

Carere et al. (2018b) found that the pathway involves two stages, as shown in Fig. 16. In the first stage, dehydrogenase DepA is used to oxidize the C3–OH group in DON to the intermediate 3-keto-DON. Then, in the second stage, another AKR DepB is adopted to reduce the 3-keto-DON back into DON, reforming the stereocenter of the molecule and producing 3-*epi*-DON. As a result, they believe that the key to the detoxification process lies in the catalytic action of DepB. This two-step differential isomerization was also reported by Hassan et al. (2017). In their work, however, the DON was treated with cell lysates of *Devosia* under aerobic conditions. The enzymes responsible for the oxidation of DON to 3-keto-DON and those involved in the reduction of 3-keto-DON to 3-*epi*-DON were physically kept separate. He et al. (2020b) also found the same pattern of DON degradation using enzymes isolated from *Devosia* strain D6-9. Therefore, this is currently considered one of the most promising ways of detoxifying DON.

Glycation can also occur at the C3 atom in DON and it has been found that the glycosylation of the C3 hydroxyl group using glycosyltransferase can convert DON into a less toxic glycoside. In 2003, Poppenberger discovered a UGT, DOGT1, in *Arabidopsis thaliana* (Poppenberger et al., 2003). DOGT1 can convert DON to 3-*O*-glucopyranosyl-4-DON by catalyzing the transfer of glucose from UDP-glucose to the C3 hydroxyl group in DON. The enzyme was also found to detoxify the acetylated derivative 15-acetyl-DON. Schweiger et al. (2010) expressed the gene encoding UDP-glycosyltransferase in *A. thaliana* (*HvUGT13248*) and found that it is responsible for the conversion of DON into DON-3-*O*-glucoside, thus reducing the toxicity of DON. The enzyme has also been found to detoxify metabolites produced by plant-pathogenic microorganisms (Schweiger et al., 2013).

Li et al. (2015) found that transgenic wheat expressing a barley UGT showed significantly higher resistance to *Fusarium graminearum*. Wetterhorn et al. (2016) further studied the role of UDP-glycosyltransferase (Os79) on the amino acid residues involved in the catalytic mechanism. It was found that His-27 activates the C3 hydroxyl group in DON and carries out a nucleophilic attack on the C1′ atom in the UDP glucose donor. Thr-291 plays a key role in the process as a catalytic acid or in locating the UDP portion during nucleophilic attack. This is similar to the UGT catalytic mechanism in other plants. It has also been found that DON can be converted to different mono-glucuronides by glucuronidation using animal liver microsomes (Schwartz-Zimmermann et al., 2017).

Another way to degrade DON is via the acetylation of site C3. Kimura et al. (1998) successfully cloned a gene from *F. graminearum*, *Tri101*, that can be used for acetylation and re-expressed it in *Escherichia coli* thus obtaining trichothecene 3-*O*-acetyltransferase. This enzyme was then used to introduce an acetyl group to the C3 atom in DON in an acetyl-CoA-dependent manner for detoxification purposes. Khatibi et al. (2011) found that the enzyme encoded by the gene *TRI201* is also able to convert DON to 3-acetyl-DON (3-ADON) with a conversion rate between 50.5% and 100.0%. In addition, Garvey et al. (2009) reported a 15-*O*-trichothecene acetyltransferase (TRI3) which they isolated from *Fusarium sporotrichioides*. Tokai et al. (2008) found that rTRI3 protein also exhibits 4-*O*-acetylation activity. In addition to 15-acetyl-DON, diacetylated derivatives were detected at high enzyme concentrations (4, 15-diacetyl-DON and, to a lesser extent, 3, 15-diacetyl-DON).

The specialized glyoxalase I from *Gossypium hirsutum* (SPG) can lower the toxicity of 3A-DON by conducting isomerization to transfer C8 carbonyl to C7 and the double-bond from C9–C10 to C8–C9. Furthermore, SPG can also recognize 15A-DON and DON. Notably, by constructing the variant SPG^Y62A^, heterogeneously expressed in *P. pastoris*, the catalytic activity of DON and its acetylated derivatives was increased by 70% (Hu et al., 2022). It has also been found that glutathione-S-transferase can block/minimize the entry of DON into the cytoplasm, thereby minimizing overall toxicity (Hassan et al., 2019; Gardiner et al., 2010).

In summary, gaining an understanding of the enzymatic pathways available for the biodegradation of DON is crucial for developing products containing microorganisms that possess this ability.

### 5.3 Application of DON-degrading enzymes in animal production

At present, there are relatively few protective effects of DON-degrading enzymes on animal production. It has been reported that some degrading bacteria such as *Devosia* sp. ANSB714, *B. subtilis* ASAG 216 can not only improve the adverse effects of DON on the production performance of pigs, but also significantly reduce the residue of DON in serum, liver, and kidney (X. Li et al., 2018; Jia et al., 2021). Tso et al. (2019) found (experimentally) that, in simulated gastrointestinal tracts in pigs and poultry, enzyme degradation reagents were more effective in reducing DON and ZEN contamination compared to the adsorbent approach. This suggests that degrading enzymes also have certain advantages in protecting the animal gut from DON damage. However, more in vivo studies are needed to elucidate the role of degrading enzymes in animal protection.

## 6 Degradation of mixed toxins using mycotoxin-degrading enzymes

Microbial enzymes have been found to be able to degrade mixtures of toxins *simultaneously*. For example, X. Wang et al. (2019a) first studied the ability of a laccase derived from *B. subtilis* (*Bs*CotA) to degrade a mixture of ZEN and AFB1. When methyl syringate was used as a mediator, the degradation rates of ZEN and AFB1 achieved using *Bs*CotA were found to be 98% and 100%, respectively. Loi et al. (2018) evaluated the ability of laccase and laccase–mediator systems from *Pleurotus eryngii* to degrade multiple mycotoxins in vitro and found that AFB1 and ZEN were simultaneously degraded by 86% and 100%, respectively.

Azam et al. (2019) used gene fusion technology to combine genes encoding ZHD and carboxypeptidase thus creating a fusion enzyme, ZHDCP. The new enzyme was subsequently shown to be able to degrade both OTA and ZEN. Moreover, OTA was completely degraded after just 30 min (at pH 7.0 and 30 °C). Qin et al. (2021c) identified, cloned, and heterogeneously expressed a new multicopper oxidase (MCO)-encoding gene, *StMCO*, from *Streptomyces thermocarboxydus*. The purified recombinant *St*MCO exhibited a characteristic blue color and cupric-ion-dependent enzyme activity. It was directly able to degrade AFB1 and ZEN without the use of a mediator. Furthermore, in the presence of ABTS, or natural mediators derived from various lignin units, the degradation rates using *St*MCO were significantly enhanced.

In addition, X. Wang et al. (2019b) found that manganese peroxidase can degrade four main mycotoxins (AFB1, ZEN, DON, and fumonisin B_1_) in the presence of dicarboxylic acid malonate. Qin et al. (2021a) further proved that the recombinant *Bs*DyP obtained by the heterologous expression of *BsDyP* gene from *B. subtilis* SCK6 could degrade different types of mycotoxins, including AFB1, ZEN, and DON, in presence of Mn^2+^. More importantly, their corresponding enzyme degradation products AFB1-diol, 15-OH-ZEN, and C_15_H_18_O_8_ have significantly lower toxicities than AFB1, ZEN, and DON.

Clearly, if the toxicity of the residual degradation products is carefully checked and found to be favorable, it may be possible to use microbial enzymes to simultaneously degrade multiple mycotoxins in food or feed. The works mentioned in this review give some insight into the approaches currently available for finding and developing new degrading enzymes that can be mass produced and used to detoxify harmful mycotoxins.

## 7 Conclusions

Mycotoxins contamination has brought great economic losses to the food and animal husbandry industries and is a serious threat to human health and social development. The use of physical, chemical, and biological adsorption detoxification to alleviate mycotoxins in food and feed is always limited. Compared with these methods, enzymatic degradation has incomparable advantages. However, research into the usage of mycotoxin-degrading enzymes in animals remains insufficient. This may be limited to the fact that the degradation mechanism of mycotoxins and the structure and toxicity of degradation products have not been fully discussed. At the same time, the optimal activity of the degrading enzyme needs to be further optimized to adapt to the acidic environment in the animal body, such as the stomach. Therefore, the practical application of degrading enzymes needs more research into the identification of mycotoxin-degrading enzymes, molecular modification, and toxicity evaluation of degradation products.

X-ray diffraction or computer-aided molecular dynamic simulation can help analyze the structural changes of degrading enzymes before and after composite substrates or products, and then better guide scholars to rational transformation of enzyme molecules. In addition, by using metagenomic technology, gene sequencing technology, and computer-assisted screening, new biological enzymes with excellent catalytic performance can be efficiently obtained from a huge gene database. Cell-free protein expression and other technologies developed in recent years provide an effective means for high-throughput screening of enzymes, which can greatly shorten the discovery cycle of new enzymes. Therefore, it is believed that in the future, mycotoxin-degrading enzymes are expected to appear in the feed and food industry to be applied to protect animal production from mycotoxin injury.

## Author contributions

**Huiying Sun:** writing—original draft preparation. **Ziqi He**, **Dongwei Xiong, Miao Long:** writing—review and editing. **Miao Long:** supervision.

## Declaration of competing interest

We declare that we have no financial and personal relationships with other people or organizations that can inappropriately influence our work, and there is no professional or other personal interest of any nature or kind in any product, service and/or company that could be construed as influencing the content of this paper.
